# Magnetically‐actuated microcages for cells entrapment, fabricated by laser direct writing via two photon polymerization

**DOI:** 10.3389/fbioe.2023.1273277

**Published:** 2023-12-19

**Authors:** Roxana Cristina Popescu, Bogdan Stefanita Calin, Eugenia Tanasa, Eugeniu Vasile, Mona Mihailescu, Irina Alexandra Paun

**Affiliations:** ^1^ Department of Bioengineering and Biotechnology, Faculty of Medical Engineering, Politehnica University from Bucharest, Bucharest, Romania; ^2^ Department of Life and Environmental Physics, National Institute for R&D in Physics and Nuclear Engineering “Horia Hulubei”, Magurele, Romania; ^3^ Faculty of Applied Physics, Politehnica University from Bucharest, Bucharest, Romania; ^4^ Center for Advanced Laser Technologies (CETAL), National Institute for Laser, Plasma and Radiation Physics, Magurelee, Romania; ^5^ Department of Physics, Faculty of Applied Physics, Politehnica University from Bucharest, Bucharest, Romania

**Keywords:** microcage-like structures, laser direct writing, two photon absorption, cells entrapment, magnetic actuation Justified, space after: 0 pt, pattern: clear (white)

## Abstract

The manipulation of biological materials at cellular level constitutes a sine *qua non* and provocative research area regarding the development of micro/nano‐medicine. In this study, we report on 3D superparamagnetic microcage‐like structures that, in conjunction with an externally applied static magnetic field, were highly efficient in entrapping cells. The microcage‐like structures were fabricated using Laser Direct Writing via Two‐Photon Polymerization (LDW via TPP) of IP‐L780 biocompatible photopolymer/iron oxide superparamagnetic nanoparticles (MNPs) composite. The unique properties of LDW via TPP technique enabled the reproduction of the complex architecture of the 3D structures, with a very high accuracy i.e., about 90 nm lateral resolution. 3D hyperspectral microscopy was employed to investigate the structural and compositional characteristics of the microcage‐like structures. Scanning Electron Microscopy coupled with Energy Dispersive X‐Ray Spectroscopy was used to prove the unique features regarding the morphology and the functionality of the 3D structures seeded with MG‐63 osteoblast‐like cells. Comparative studies were made on microcage‐like structures made of IP‐L780 photopolymer alone (i.e., without superparamagnetic properties). We found that the cell‐seeded structures made by IP‐L780/MNPs composite actuated by static magnetic fields of 1.3 T were 13.66 ± 5.11 folds (*p* < 0.01) more efficient in terms of cells entrapment than the structures made by IP‐L780 photopolymer alone (i.e., that could not be actuated magnetically). The unique 3D architecture of the microcage‐like superparamagnetic structures and their actuation by external static magnetic fields acted in synergy for entrapping osteoblast‐like cells, showing a significant potential for bone tissue engineering applications.

## 1 Introduction

More than 60 years ago, Richard Feynman was imagining small robots the size of the cells in the human body, that were able to interfere with them when needed (Feynman, 1959). Nowadays, with the development of micro‐/nano‐fabrication techniques, the design and fabrication of such robots became possible (Cabanach et al., 2020; Jin et al., 2020). In this context, the laser micro‐ and nano‐patterning approach has proved a great ability in the controlled production of micro‐/nano‐structures with sub‐micron resolution (Yang et al., 2019). Laser‐generated micro‐/nano‐structures for guiding the cell adhesion, growth and differentiation (Paun et al., 2018a; Babaliari et al., 2018; Alshehri, 2021; Bakhtina et al., 2021; Shivakoti et al., 2021) varied from modified topographies (Babaliari et al., 2018; Shu et al., 2021) to controlled geometries (Martínez-Calderon et al., 2020; Alshehri, 2021) and, most recently, to complex 3D architectures (Paun et al., 2018b; Czich et al., 2020; Rengarajan et al., 2020) etc.

One of the most advanced laser‐assisted technologies is Laser Direct Writing via Two‐Photon Polymerization (LDW via TPP), which enables the fabrication of complex 2D and 3D structures with resolution of 90 nm in regard to lateral spatial features. LDW via TPP is considered to be a high‐resolution 3D printing technique, with no limitations regarding the architecture of the obtained structures and with high accuracy and reproducibility of the design. To date, LDW *via* TPP was extensively used in the biomedical field to print various 3D biomimetic configurations for tissue engineering applications (Paun et al., 2018a; Hauptmann et al., 2019; Paun et al., 2020a), for controlled wettability of surfaces with antifouling properties (Paun et al., 2021), for micro‐/nano‐topographies for restrained cell attachment (Paun et al., 2020b; Song et al., 2021), for drug delivery systems via controlled release (Cabanach et al., 2020) as well as for electromagnetic stimulation of cells (Paun et al., 2019; Vaithilingam et al., 2019).

Within the above wide scientific context, there are emerging niche applications that require a more accurate control of the cells behavior. For example, restraining the cells attachment to certain surfaces, directing cells growth and inducing cells differentiation are some of the main goals in designing complex 3D architectures for biomedical applications (Liao et al., 2020; Paun et al., 2020a; Sharaf et al., 2022). Until present, different cells entrapment techniques have been extensively studied regarding their ability to immobilize cells in different pathologies, such as cancer metastasis (Ju et al., 2020; Preciado et al., 2021), microbial infections (Li et al., 2021a) or inflammation (Schmidt and Wittrup, 2009), etc., as well as for tissue engineering applications (Khetan et al., 2009; Guvendiren and Burdick, 2012) or for “cell delivery” applications (Gatto et al., 2023).

Cells entrapment techniques include physical encapsulation in polymeric beads, such as microgels (Zhou et al., 2018; Veernala et al., 2021) or alginate beads (Shao et al., 2020; Hasturk et al., 2022), penetration and attachment of cells into porous 3D scaffolds (Wu et al., 2020; Czosseck et al., 2022) or fiber‐based matrices (Matera et al., 2019; Davidson et al., 2021; Sahu et al., 2021), bioreactors based on porous membranes (Skrzypek et al., 2017; Bose et al., 2020), films made of super‐adhesive materials (Suneetha et al., 2019; Nagano et al., 2021) and antibody‐conjugated magnetic beads (Xu H et al., 2011; Nath et al., 2015). These devices can be obtained using innovative technologies such as 3D printing (Agarwal et al., 2020; Dey and Ozbolat, 2020), photolithography (Tricinci et al., 2015; Larramendy et al., 2019; Tenje et al., 2020), electrospinning (Canbolat et al., 2011; Zussman, 2011; Ang et al., 2014), emulsion methods to obtain polymeric droplets (López et al., 1997; Chaemsawang et al., 2018; Qu et al., 2021), surface coating technologies (Yoo et al., 2011), sol‐gel encapsulation (Kamanina et al., 2022), template‐assisted techniques (Khademhosseini et al., 2006), etc. The cells are kept inside the device through physical immobilization (Zhou et al., 2018; Shao et al., 2020; Veernala et al., 2021; Hasturk et al., 2022), extracellular‐matrix‐like adherence (Rao and Winter 2009), specific antigen‐antibody recognition (Roupioz et al., 2011; Boulanger et al., 2022), barrier containing (Spagnolo et al., 2015; Larramendy et al., 2019; Li et al., 2021b) and external stimuli‐activated entrapment (Fu et al., 2008; Long et al., 2020), etc. The entrapment of cells can be switched on and off through enzymatic control (Chen et al., 2003), pH variations (Kocak et al., 2017), barrier containing (Seifan et al., 2017; Larramendy et al., 2019; Gatto et al., 2023) or radiation switch (Fu et al., 2008; Long et al., 2020).

The development of reconfigurable micro‐devices that respond to changes in the environment is an additional step towards obtaining actuated micro‐robots with applications in medicine (Jin et al., 2020; Li et al., 201a). Using such gadgets for cell immobilization can advance their utilization in tissue repair (Sharaf et al., 2022), microsurgery (Fu et al., 2008; Jin et al., 2020) or on site diagnosis (Li et al., 2021b).

Cell trapping is a topic that gained significant attraction in the last decade because it provides a way of analyzing physiological dynamics of individual cells in order to provide better understanding of characteristics, such as cells metabolism. However, cell trapping is highly dependent on the methods and devices used to achieve this. Most methods involve some form of microfluidic device and/or architecture (Nilsson et al., 2009; Park et al., 2010; Shah et al., 2014; Deng, et al., 2019; Deng et al., 2020; Luan et al., 2020).

In this study, we propose and validate a proof of principle regarding laser‐assisted fabrication of complex microcage‐like structures with unique 3D architecture and composition, with the aim to facilitate the entrapment of single or of low number of cells within the structures.

The 3D architecture and the composition of the microcage‐like structure had the role to provide an adherent environment for the cells to attach‐to be entrapped‐ and to grow.

The structures were fabricated by LDW *via* TPP of a biocompatible nanocomposite material with superparamagnetic properties, in the shape of 3D microcage‐like structures able to entrap human cells through static magnetic field activation. The microcages were obtained by mixing of IP‐L780 photopolymer with magnetic iron oxide nanoparticles (MNPs). The MNPs have been previously coated with polyethylene glycol shells (Popescu et al., 2020) in order to improve their dispersion in the polymer matrix. Starting from this composite material, we fabricated, tested and validated *in vitro* an innovative system based on microcage‐like structures that were able to entrap osteoblest‐kile cells through magnetic activation, with applications in tissue engineering of bone. The novelty of the study consists in both the composition and the architecture of the proposed structures, as well as in proving their functionality *in vitro* biological environments, i.e., in osteoblast‐like cell cultures. In addition, we also found that the cell entrapment ability of these structures could be triggered by their exposure to external static magnetic fields.

As far as we know, until present no cell‐entrapment studies were carried out using structures fabricated by LDW via TPP. In our study, the use of this laser additive manufacturing technique at microscopic level was chosen in order to further improve the results of the above‐mentioned approaches. This was possible because LDW via TPP allows to obtain complex, arbitrary 3D microstructures that are controllable in shape and size, which makes them very suitable for systematic studies (Calin and Paun, 2022). High reproducibility and ease of manufacture are important benefits as well, albeit often overlooked. Moreover, as shown in our paper, various stimuli can be incorporated at a local level, in order to better isolate factors that influence a single cell’s behavior and study its dynamics in a way that cannot be achieved using bulk/stochastic analysis. The shape and size of individual cell traps account for several main factors (Calin and Paun, 2022). One of these factors is the structure’s stability, in relation to the aspect ratio (height to cross section). LDW via TPP results in polymeric structures that are defined, among other things, by a polymerization degree. This polymerization degree determines mechanical strength, especially during the developing process. During sample development and shortly after, structures are significantly softer and more flexible. This happens because of two reasons, mainly: the stochastic character of the underlying fabrication method (random polymeric chains form under laser irradiation, which results in some monomer, solvent and photoinitiator molecules to be trapped throughout the volume of the resulting polymer) and the development method (the immersion in PGMEA for a period of time allows for solvent molecules to migrate throughout the porous polymeric structures until the structure is dried, affecting its mechanical characteristics). Therefore, the shape of the main pillar provides better mechanical strength, which in turn allows the structures to be taller. This is very important for the sample’s developing stage, as the surface tension of the evaporating solvent may permanently bend microstructures, if they do not have the appropriate characteristics.

## 2 Materials and methods

### 2.1 Structures design

The design of the microcage‐like structures was based on. stl files and using DeScribe (Nanoscribe proprietary software). The stl file represents the outer surface of the structure. Then, DeScribe software was used to generate the laser path throughout the whole volume of the designed structure, as well as to determine the laser writing parameters such as the laser writing speed and the laser power.

The microcage‐like structures were fabricated in a layer‐by‐layer manner. The laser path used for polymerization of the photopolymerizable material was defined in two parts. The first part represents the shell of the structure, which is formed by multiple layers both in Z direction, as well as towards the center of the structure. The layers of the shell follow the contour of the outer surface of a microcage. The second part addresses the inner volume of the structures, where we used parallel lines (hatching) to polymerize the inner parts of the photopolymerizable material. The distance between neighboring lines in the Z direction was set to 2 μm, while the distance between neighboring lines in the XY plane was set to 1 μm. These values were chosen to accommodate the magnetic nanoparticles that were dispersed throughout the IP‐L780 photopolymer, as potential nanoparticle clusters encountered by the laser beam inside the photopolymer could result in local microexplosions due to near field intensification effects, which drastically affect the morphology of the laser‐imprinted structures. The diameter and the height of a microcage‐like structure were set to of 30 μm and 80 μm respectively.

### 2.2 Structures fabrication

The microcage‐like structures were fabricated using LDW *via* TPP on IP‐L780 photopolymer mixed with superparamagnetic nanoparticles (MNPs) that were homogeneously dispersed throughout its volume. The MNPs were previously synthesized and characterized using a two‐step room temperature co‐precipitation method and then encapsulated in polyethylene glycol of molecular weight 6,000 Da, in order to improve the MNPs dispersion in the photopolymer (Popescu et al., 2020).

The Poly (ethylene glycol) used for the encapsulation of the iron oxide nanoparticles was acquired from Sigma Aldrich‐ Merck (product number 81255 BioUltra 6,000, CAS Number 25322‐68‐3). The product specification sheet of this commercial product, available on the company’s website, states that the molecular mass is between 5,000‐7,000 and was calculated from the hydroxyl value. This product was chosen due to the low impurities concentration and its destination for biological applications, as stated by the company. Sigma Aldrich‐ Merck is a well‐known company in the field of chemical substances, thus the technical information on the products is highly reliable. A higher molecular weight of the polymer might increase the hydrodynamic properties of the nanosystem and a lower molecular weight polymer might increase the aggregation of the particles. We have previously developed the method to obtain the PEG 6000‐ encapsulated iron oxide nanoparticles, which have proved the best reproducibility, hydrodynamic properties, as well as biocompatibility for biological structures (Popescu et al., 2020; Popescu et al., 2021; Popescu et al., 2022a; Popescu et al., 2022b; Popescu et al., 2022b; Tudor et al., 2023). The MNPs with an average physical diameter of 12.82 ± 2.73 nm were previously synthesized and characterized using a two‐step room temperature co‐precipitation method and then encapsulated in polyethylene glycol of molecular weight 6,000 Da (Sigma Aldrich‐ Merck, Darmstadt, Germany), in order to improve the MNPs dispersion in the photopolymer, due to the core‐shell morphology of the MNPs (Popescu et al., 2020). The resulted MNPs have a single crystalline phase of magnetite with spinel structure (Popescu et al., 2020).

A complete characterization of the magnetic beads has been provided in the following literature: (Popescu et al., 2020). The purpose of the present study is to characterize the IP‐L780/MNPs composite, which was done through Scanning Electron Microscopy and Energy Dispersive X‐Ray Spectroscopy (Figure 7). The MNPs with an average physical diameter of 12.82 ± 2.73 nm were previously synthesized and characterized using a two‐step room temperature co‐precipitation method and then encapsulated in polyethylene glycol of molecular weight 6,000 Da (Sigma Aldrich‐ Merck, Darmstadt, Germany), to improve the MNPs dispersion in the photopolymer, due to the core‐shell morphology of the MNPs (Popescu et al., 2020). The resulted MNPs have a single crystalline phase of magnetite with spinel structure (Popescu et al., 2020).

The composite superparamagnetic photopolymerizable material was obtained by a simple physical mixing of the MNPs at a concentration of 4 mg/mL into IP‐L780 photopolymer, by ultrasound dispersion (Ultrasonic Homogenizer 300 V/T, Biologics Inc. Manassas, Virginia United States, at 75 W, for 3 min, 10 pulses). The concentration of 4 mg/mL of the superparamagnetic MNPs that were dispersed in the IP‐L780 photopolymer was selected based on our recent finding (Paun et al., 2019) that indicated that this particular MNPs concentration enables the laser‐fabrication of 3D complex microstructures that poses good mechanical stability and that also provide the faster mineralization of osteoblast‐like cells seeded on those structures, as compared to MNPs concentrations below 4 mg/mL. The above‐mentioned previous study also indicated that MNPs concentrations above 4 mg/mL did not allow for structure fabrication via LDW via TPP, due to MNPs agglomeration due to poor MNPs dispersion in the photopolymer, which caused localized absorption of the incident laser beam, which further leaded to local micro‐explosions of the photopolymerizable material, resulting in mechanical disintegration of the microstructures.

The laser writing system used for fabricating the microcage‐like structures was Nanoscribe^®^ Photonic Professional. The incident laser beam was generated by an Er‐doped fiber laser that delivered 150 fs laser pulses with a frequency of 80 MHz and a maximum average laser power of 120 mW. The central wavelength was 780 nm. The main physical phenomenon responsible for the laser‐matter interaction was two photon absorption. The photopolymer is transparent to the central wavelength of the incident beam and the polymerization is mediated by a photoinitiator that has high absorption for the second harmonic of 390 nm (Paun et al, 2018a).

In order to obtain high spatial resolution of the laser‐imprinted structures, the system was based on a stationary laser beam and moving sample. Namely, the sample was moved using a dual stage system. The first positioning system was represented by a motorized translation stage with micrometer positioning accuracy, complemented by a linear stage for optical focusing of the laser beam. This stage was used for coarse positioning of the sample. The second system was represented by a high resolution XYZ piezo stage that was used for fabricating the microcage‐like structures. For this, the photopolymerizable material was drop‐casted on 170 μm thick glass slides that were inserted in a metallic sample holder. The laser beam was then focused into the photopolymerizable material using a ×63 microscope objective. Given the high morphological complexity of the microcage‐like structures, the laser writing process was performed in a reversed geometry, in order to allow the polymerization throughout the whole i.e., complex laser path through the photopolymerizable material, while avoiding a multiple passing of the laser beam through already polymerized regions (Paun et al, 2020a).

### 2.3 Characterization

#### 2.3.1 Scanning electron microscopy and energy dispersed X ray spectroscopy

The microcage‐like structures were characterized by scanning electron microscopy (SEM) that provided information on the morphological and dimensional characteristics of the samples. SEM investigations were performed by using a Quanta Inspect F microscope (FEI Company) equipped with an energy dispersive X‐ray spectrometer (EDX). The micro‐cages were visualized in absence and in presence of the MNPs. The samples were gold‐sputtered (∼10 nm) prior to SEM investigations.

#### 2.3.2 Enhanced dark field microscopy

Enhanced dark field images were acquired using the CytoViva^®^ System (Auburn, AL, Unites States) module for fluorescent microscopy. In this configuration, taking advantage of a condenser with a patented shape, the microcage‐like structures were illuminated at a high oblique angle (the direct illumination did not enter in the objective lens). In this way, we were able to obtain images with an improved signal‐to‐noise ratio, i.e., bright spots from the scattered light by the sample on a very dark background.

A mercury lamp (Lumen200, Prior Scientific Instruments Ltd., United Kingdom) illuminateed the darkfield condenser (cardioid‐shaped, oil immersed) through a liquid‐core optical fiber, to diminish the thermal noise. Between the mercury lamp and the condenser, an excitation filter was inserted i.e., FITC (fluorescein isothiocyanate). The images were formed through an ×60 oil immersed objective, on a cooled EXiBlue monochrome CCD (QIMAGING Corporation, Canada, 1392 × 1040 pixels, recording 15 fps at maximum resolution, 6.45 × 6.45 µm pixel size. The emission filter was placed between the microscope objective and the CCD. To obtain 3D images, the microcage‐like structures were scanned along their height, with a motorized stage (NanoScanZ, Prior Scientific Instruments Ltd., United Kingdom, 100 nm step size, 114 × 75 mm travel range). Several cross‐sections were acquired, at a distance of 100 nm between them.

Two sets of images were consecutively recorded, by Z‐scanning of the structures along the 0Z‐axis (propagation axis, perpendicular to the glass slide plane), as follows: one set was acquired by illuminating the3D structures through the excitation filter and the other set was obtained by illuminating the structures with “white” light from the mercury lamp (without any excitation filter in the optical path). In the first case, fluorescent Z‐stacks images of the structures were recorded. In the second case, we recorded Z‐stacks containing the scattering light from the MNPs. The used ×60 oil immersion microscope objective provided a resolution of 107.5 nm in the XY plane; its numerical aperture was set to 0.95 for fluorescent Z‐stacks images; for Z‐stacks images recorded in white light, it was set to its minimum value of 0.65.

For a given area of the sample, fluorescent Z‐stack of the structures was acquired by setting the exposure time at 50 m for each cross‐section. Setting the focalization distance unchanged in the experimental setup, the same step size and cross‐sections number (50), the second Z‐stack was recorded in white light, by setting the exposure time for one cross‐section at 3 m.

The preprocessing procedures included point spread function generation and deconvolution for fluorescent Z‐stacks (using standard codes provided by producer, under ImageJ), while for “white” light Z‐stacks, the built‐in specific plugin “just locate nanoparticle” was run (Cytoviva 3‐D, 2023).

### 2.4 Cell culture

MG63 osteoblast‐like cells (CLS, Heidelberg, Germany) were cultured in Dulbecco’s Modified Eagle Medium (DMEM, Pan Biotech, Aidenbach, Germany), supplemented with 10% FBS (Pan Biotech, Aidenbach, Germany) and 0.1% Penicillin‐ Streptomycin (Pan Biotech, Aidenbach, Germany), in standard conditions of temperature and humidity (37°C, 5 ± 1% CO_2_, 95% humidity).

Prior to cell seeding, the microcage‐like structures were sterilized during 1 h UV light exposure and then washed several times with complete cell culture medium, in order to eliminate any possible residues. Following this, 30,000 cells/25 µL were seeded onto each structure and allowed for about 15‐20 min to lay down and start attaching to the substrate. Afterwards, some fresh complete culture medium was slowly added to fill in the culture dishes. The cells were cultured for another 24 h in standard conditions of temperature and humidity, in absence or presence of static magnetic fields (+SMF), respectively. For this, the microcage‐like structures were placed in the vicinity of cubic 1.29–1.32 T Neodymium magnets, having a maximum energy of 42 MGOe, 588 N strength and coercive field strength of about 12 kOe.

### 2.5 Samples preparation and imaging by scanning electron microscopy

Following incubation in presence/absence of SMF, the cell culture media was removed and the cell‐seeded microcage‐like structures were gently washed three times using PBS. Afterwards, the cells were fixed during 1 h using 2.5% glutaraldehyde in PBS. After fixation, the cells were dehydrated using ethanol (Merck, Darmstadt, Germany) solutions of successive concentration (70%‐100%), followed by ethanol‐hexamethyldisilazane (Merck, Darmstadt, Germany) (50‐50, 25‐75, 0%‐100%). The cell‐seeded mirocage‐like structures were coated with 10 nm gold and the SEM analysis was performed using the FEI Quanta Inspect F Scanning Electron Microscope (Hillsboro, OR, United States).

### 2.6 Cell scoring and statistics

Following the SEM image acquisition, a manual cell counting was performed in order to score the cells entrapped in microcages and cells situated in the exterior of the microcages. For this, at least 100 cells were scored for each category (depending on the cell attachment rate, up to 500 cells/category). A total number of cells relative to the surface of the microcages was also calculated for each experimental condition. For this, the total microcage area was calculated using scanning electron microscopy images. Then, the total number of cells counted for each experimental condition was divided to the obtained area (in mm2). The experiments were done in triplicate and the results were shown as mean ± SEM (standard error of mean). For the statistical analysis, a Student’s *t*‐test was performed, using the Microsoft Excel implemented function, where *p < 0.05, **p <= 0.01 and respectively ***p <= 0.001.

The results are presented as effective cell numbers, meaning that we effectively counted the cells on each category of samples/experimental condition, namely, 1) microcage‐like structures made of IP‐L780 photopolymer alone in absence of SMF (IP‐L780), 2) iPL+SMF: microcage‐like structures made of IP‐L780 alone in presence of SMF (IP‐L780+SMF), 3) microcage‐like structures made of IP‐L780/MNPs composite without SMF (IP‐L780/MNPs), 4) microcage‐like structures made of IP‐L780/MNPs composite with SMF (IP‐L780/MNPs+SMF). We had at least two samples for each category. Thus, we counted 330 cells in total for IP‐L780, 200 cells for IP‐L780+SMF, 320 cells for IP‐L780/MNPs, respectively 540 cells for IP‐L780/MNPs+SMF. The total number of cells counted for each sample was divided into categories such as cells in cages, cells on the exterior of the cages and cells at the bottom of the cages. We had particular interest in the cells inside the cages and on the exterior of the cages because the glass substrate can be easily replaced by a non‐adherent material to sustain the microcage‐like pillars. The errors are obtained by applying standard deviation to the number of cells for each repetition. Thus, the graphs represent effective cell numbers for each experimental condition. Additionally, we were able to calculate a ratio between the total number of cells counted for each experimental condition and the surface covered by microcage‐like pillars (which was 0.036 mm^2^), parameter which can also indicate the cell viability, because the seeded cell number was the same for all structures (namely 30,000 cells). Student’s *t*‐test statistical analysis was performed in order to compare the significance of the data for each experimental condition.

## 3 Results

**FIGURE 1:**
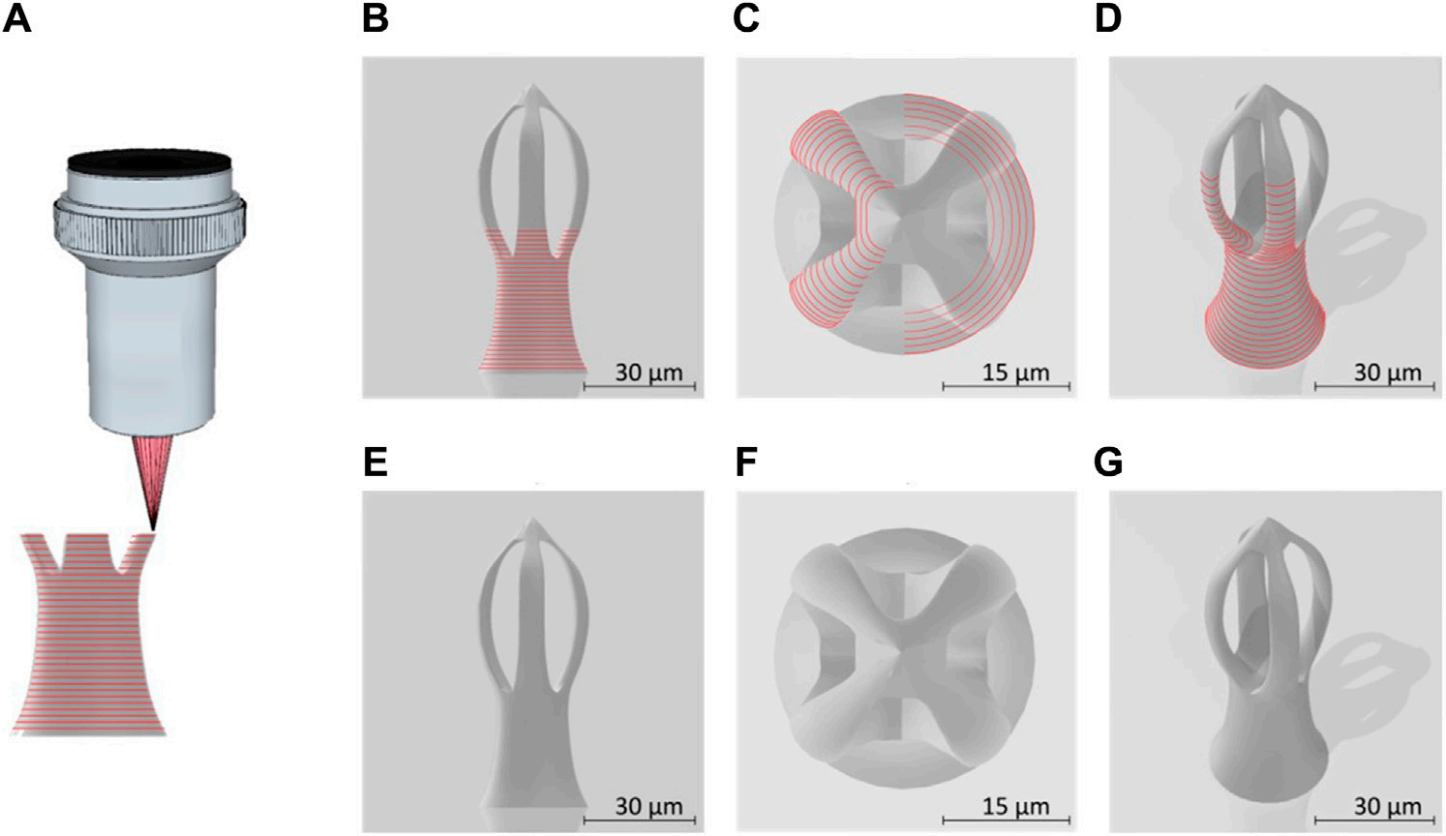
**(A)** Schematic lateral view of the laser beam focused into the photopolymer through a microscope objective, for “imprinting” microcage-like structures through laser direct writing via two photos polymerization (LDW via TPP) process; **(B)** lateral view of micro-cage design; **(C)** top view of a designed microcage; **(D)** tilted view of a designed microcage; the red lies in **(A–D)** illustrate the external path followed by the laser beam focused into the photopolymer; **(E–G)** represent the lateral, top and inclined views of a microcage design without showing the laser path throughout the structure’ volume.

Figure 1 shows the 3D design of a microcage‐like structure indicating the laser path on the surface of the structure. The base of the structure is circular, has a diameter of 30 μm and its height extends to 80 μm. While the. stl file was defined without using physical units, the DeScribe software was used to properly scale the 3D file, i.e., for transforming all the coordinates into micrometers in the cartesian system of the translation stages, as well as for defining the laser paths (slicing and hatching). A side view (Figures 1B, E) shows the layer‐by‐layer design, while top and inclined views show the contour of the surface (Figures 1C, D, F, G). In Figure 1B, it can be seen the laser path on the surface of the base of the structure, Figure 1C shows t the surface laser path on the upper part of the structure, while Figure 1D shows the laser path on the surface of the base and partially on the top of the structure.

**FIGURE 2:**
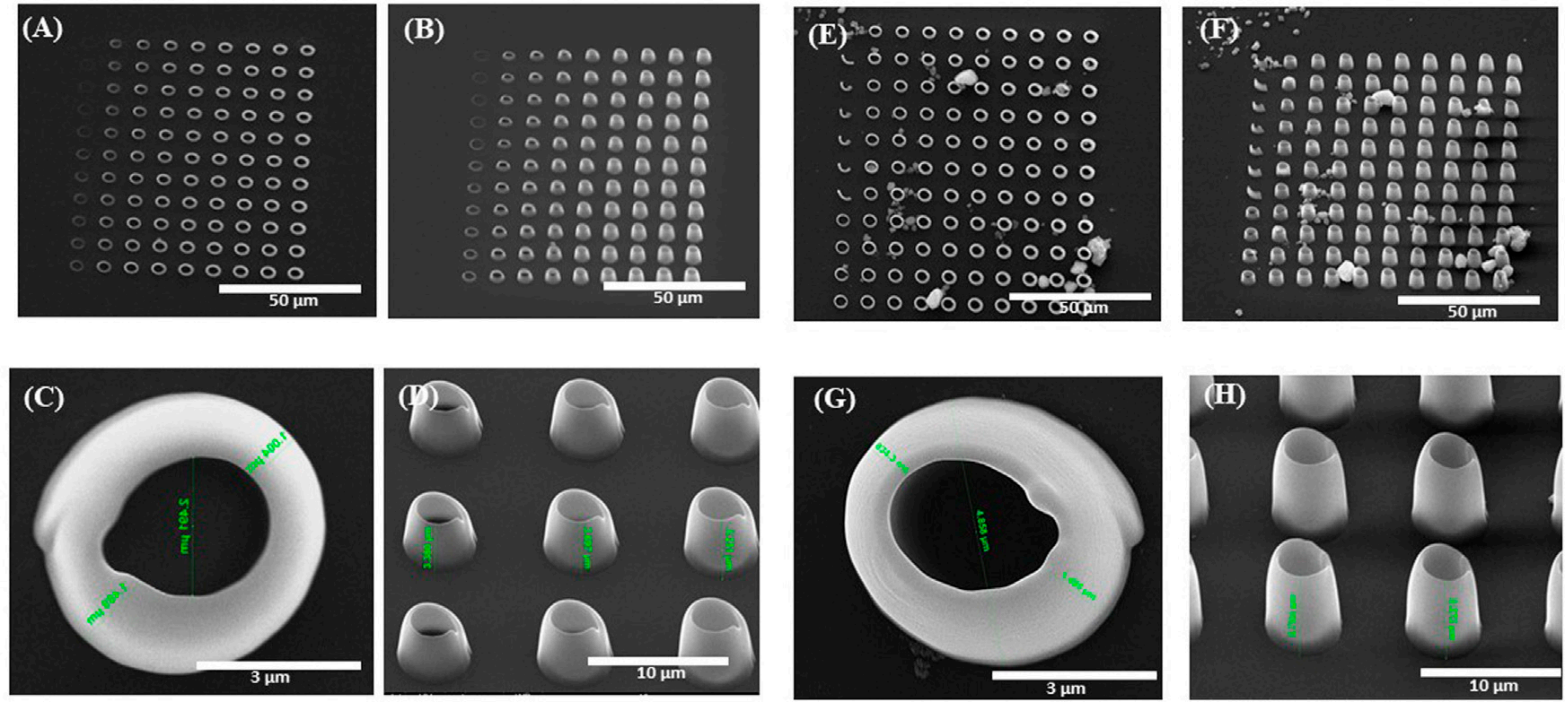
Scanning electron micrographs of 3D vertical microtubes fabricated by laser direct writing via two photon polymerization (LDW via TPP) of IP-L780 photopolymer **(A–D)** and IP-L780/MNPS composite **(E–H)**. The arrays of microtubes from **(A,B)** and **(E,F)** were obtained using different scanning speeds and laser powers. In **(A,B)** and **(E,F)** from left to right: laser power increased from 24 mW to 45.6 mW with an incremental step of 2.4 mW; from top to bottom: laser writing speed decreased from 95 μm/s to 50 μm/s with an incremental step of 5 μm/s Figure 2 **(A,C,E,G)** shows top views and Figure 2 **(B,D,F,H)** shows tilted views of the microtubes.

Figure 2 shows the parameterization map used to determine the optimal laser writing parameters (laser power and writing speed) for imprinting the 3D microcage‐like structures with highest achievable spatial accuracy. In the case of LDW via TPP process in our experimental conditions, these parameters were chosen so that the *irradiation dose* (quantity of light per unit volume) did not exceed a threshold where micro‐explosions occurred via effects such as near field intensification, solvent evaporation, and others, while also obtaining an appropriate volumetric polymerization degree and a relatively short time duration for structures fabrication (i.e., in our case, the time requested for fabricating a microcage‐like structure was of the of the order of several minutes). The irradiation dose is determined by both the laser power and the writing speed. In our experimental conditions, the parameterization maps were used to determine the optimal writing parameters that allowed the polymerization of the IP‐L8708/MNPs composite material, without producing local microexplosions, which (as also state above) could have been determined by near‐field effects of the incident laser beam interacting with clusters of magnetic nanoparticles within the IP‐L780 non‐polymerized (i.e., viscous) material, In the parameterization map, we selected to imprint elements having a vertical tubular shape, so that we can accurately analyze the morphological quality of structures to be imprinted in 3D. The microtubes were mechanically resistant to the surface tension of the evaporating solvent and therefore the developing process had a reduced (if any) influence over the parameterization results. The tests were run on microtubes fabricated by IP‐L780 photopolymer alone and, in parallel, on microtubes made by IP‐L780/MNPs composite, for comparative purposes. The experimental results presented by the SEM micrographs from Figure 2 indicated a similar energy‐dose response from both IP‐L780 and IP‐L780/MNPs composite (i.e., comparable shapes and sizes of the microtubes, regardless the presence or absence of the MNPs in the polymerized material). The differences observed between Figures 2A, E were the result of different optical focusing on the surface of the glass substrate. More precisely, the top view shows similar shape and size of a microtube, while side views show different heights, which indicates that the energy dose response of the photopolymer is similar with that of the photopolymer mixed with magnetic nanoparticles, but, in the case of IP‐L780/MNPs composite, the laser beam was focused slightly above the surface of the glass substrate.

In this study, the major focus concerns the ability of the microcage‐like structures to entrap single or low number of cells, while limiting the cells interconnections between adjacent structures or between structures and the glass substrate. Thus, the geometry and size of the microcage‐like structures was designed to limit the interactions with the cells from the glass substrate. Consequently, we set the height of the base of the microstructure to 80 μm, which is high enough to hinder the cells attached within the microcage‐like structures to interconnect with those from the glass substrate. The base diameter of the microcage‐like structure was quite large (i.e. 30 μm) that ensured a strong attachment of the microstructure to the glass substrate. The shape and the dimensions of individual microcage‐like structures from the top of the bases were selected mainly for cells to be able to easily migrate inside a trap, but also have enough room to form subsequent connections with other cells inside neighboring cages, while isolating them from cells on the substrate. The diameter of a “microcage” was set to 30 µm and the height to 40 µm. These dimensions were selected for the structures to be as close as possible to the typical dimensions of a cell (so that the “microcage” can accommodate a single cell, or‐at most‐very few cells). The distance between adjacent microcage‐like structures was set to 70 μm, so that it allows the connection between cells on neighboring structures. Overall, these dimensions and the geometries were selected for supporting the main objective of the study: to provide evidence if and to what extend the Static Magnetic Field (SMF) actuates the IP‐L780/MNPs superparamagnetic microcage‐like structures, regarding the following two points: on the one hand, to see if the SMF reduces the number of interconnections between cells attached on the structures, while, on the other hand, to check if the SMF increases the number of cells that are entrapped inside the microcage‐like structures.

**FIGURE 3:**
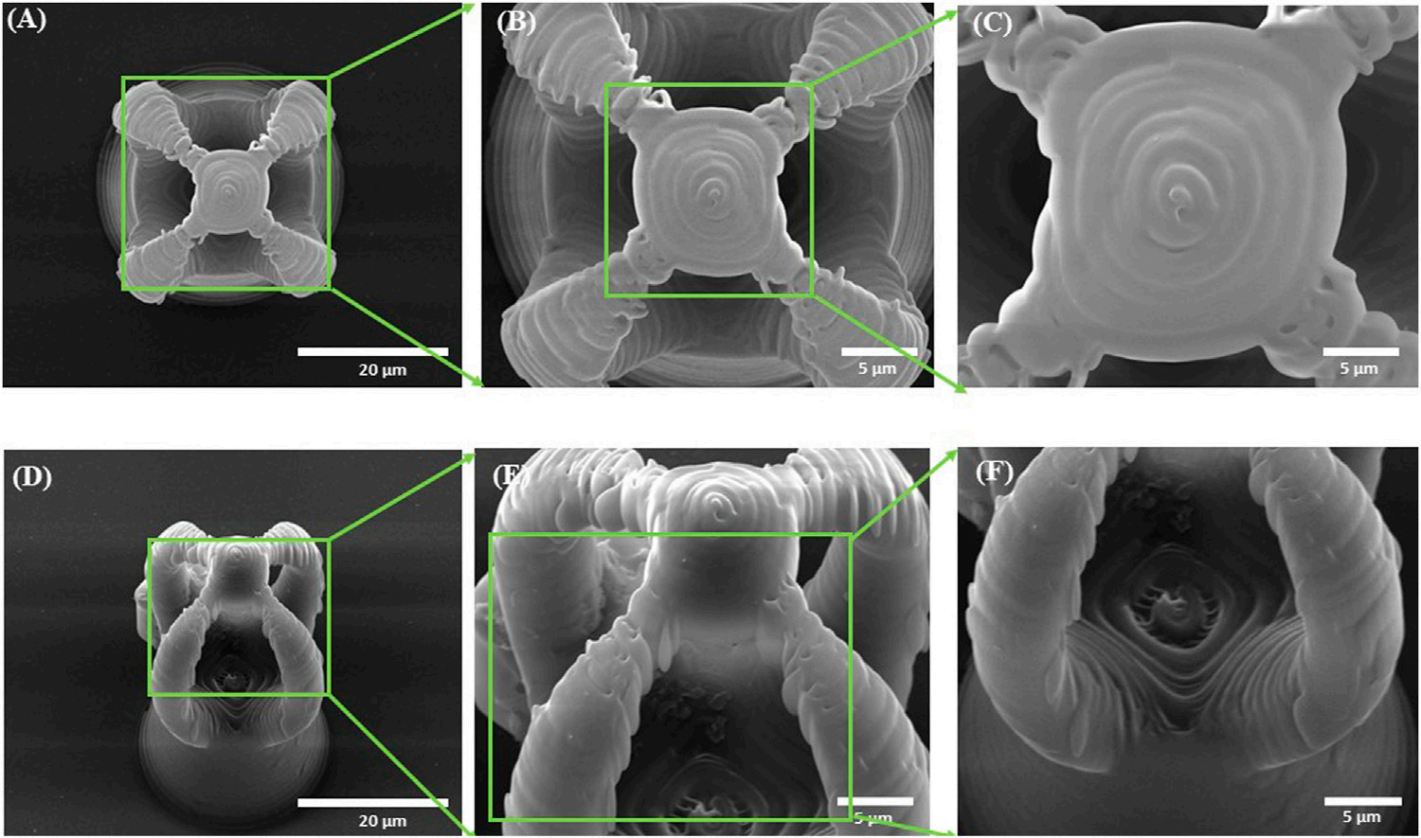
Scanning electron micrographs of microcages fabricated by LDW via TPP of IP-L780 photopolymer: **(A)** top view; **(B)** inset from **(A)**; **(C)** inset from **(B)**; **(D)** tilted view; **(E)** inset form **(D)**; **(F)** inset from **(E)**.

**FIGURE 4:**
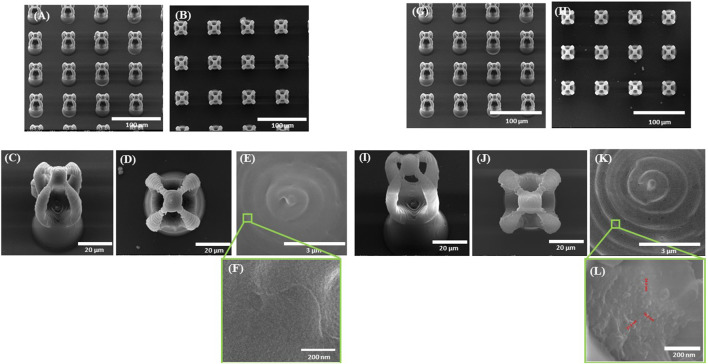
Scanning electron micrographs of microcage-like structures fabricated by LDW via TPP of: **(A–F)** IP-L780 photopolymer: **(G–L)** IP-L780/MNPs composite; **(A,G)** tilted view of arrays of microcages; **(B,H)** top views of arrays of microcages; inset from **(C,D)** tilted views; **(J)** top views; **(E,K)** insets from **(D)** and **(J)** respectively; **(F)** and **(L)** insets from **(E)** and **(K)** respectively.

The morphology of the microcage‐like structures obtained following the design and parameterization steps is detailed in Figures 3, 4. Scanning electron microscopy (SEM) was a helpful tool for highlighting the complex and unique architectural features of the structures. On the structures made of IP‐L780 photopolymer there can be clearly seen some sub‐micrometric structural elements that corresponded to the spiral‐shaped lines imprinted in the photopolymer by the focused laser beam and according to the design (Figures 3B, C, E, F). These submicrometric features provided to the surface of the microcages a certain roughness, with possible points for cells to attach on the structure. It has been previously shown that patterned surfaces enable many points for the cells to adhere (Majhy et al., 2021), as a result of enhanced surface energy. This possibility will be discussed in more detail in the paragraphs related to the results obtained *in vitro*.

Following the incorporation of magnetic nanoparticles (MNPs) in the IP‐L780 photopolymer there were no obvious changes of the micrometric morphology of the resulting structures (Figures 4A–D). However, at higher magnifications, the SEM micrographs revealed the presence of submicronic nanoparticle aggregates (Figure 4L). In comparison to the smooth surface of the microcage‐like structures made by IP‐L780 photopolymer alone (Figure 4F), at nanometric level the microcage‐like structures that contained MNPs exhibited surface features within the nanometers range which most likely corresponds to MNPs and MNPs aggregates. These nanometric features observed exclusively on the microcage‐like structures made of IP‐L780/MNPs composite provide to the resulting structure a dual (micro‐nano‐) ‐scaled morphology (Figure 4L).

**FIGURE 5:**
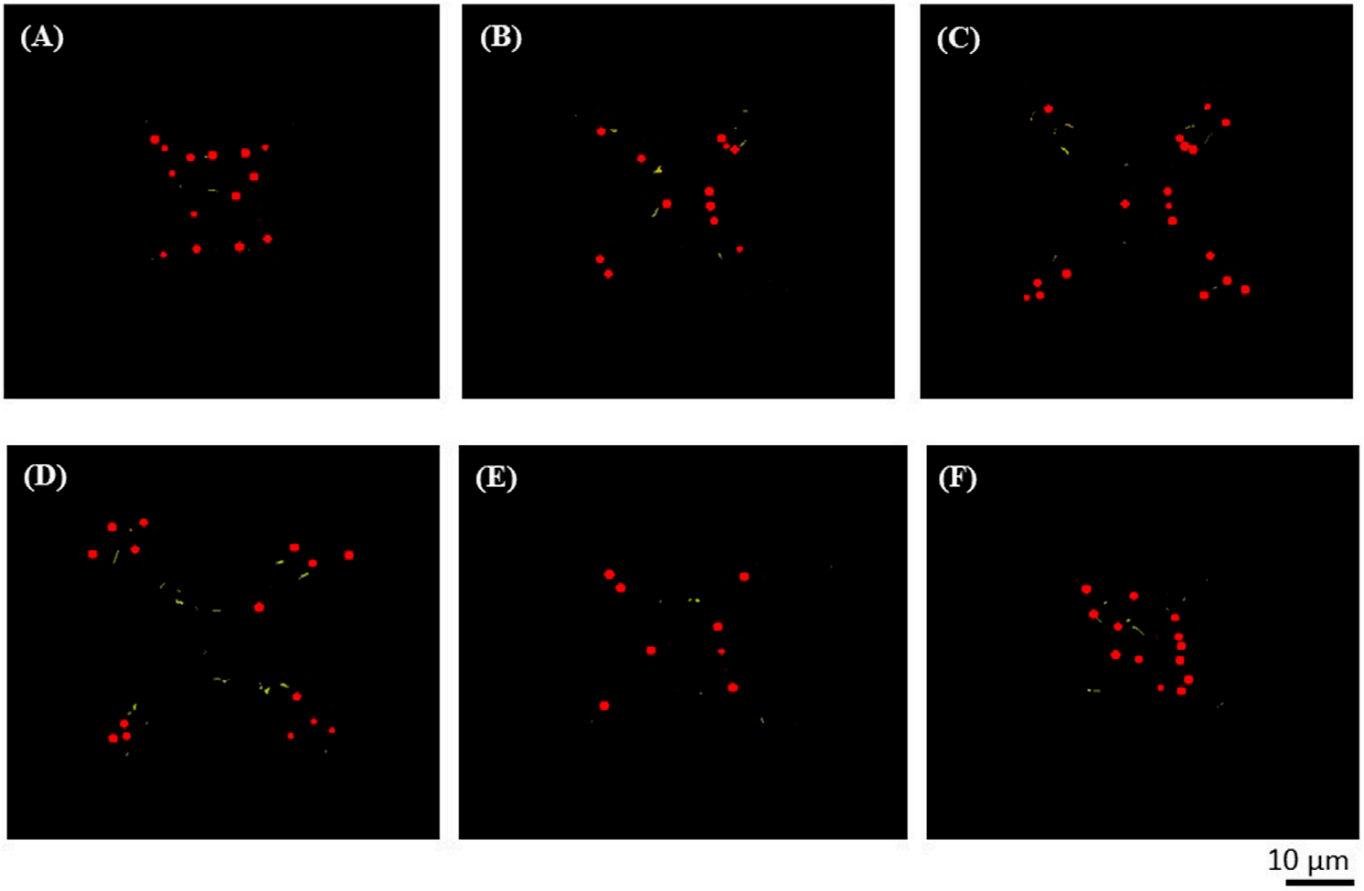
Top views obtained by enhanced dark field microscopy of a microcage-like structure (as the one displayed in Figure 4J. The photopolymer autofluorescence is colored in yellow and the centers of MNPs and MNPs aggregates are colored in red (false colors). From **(A)** to **(F)** the images were recorded at different focusing depths on the microcage-like structure: **(A)** focus on the top of the micro-cage; **(B–F)** incremental focusing depths inside the structure.

**FIGURE 6:**
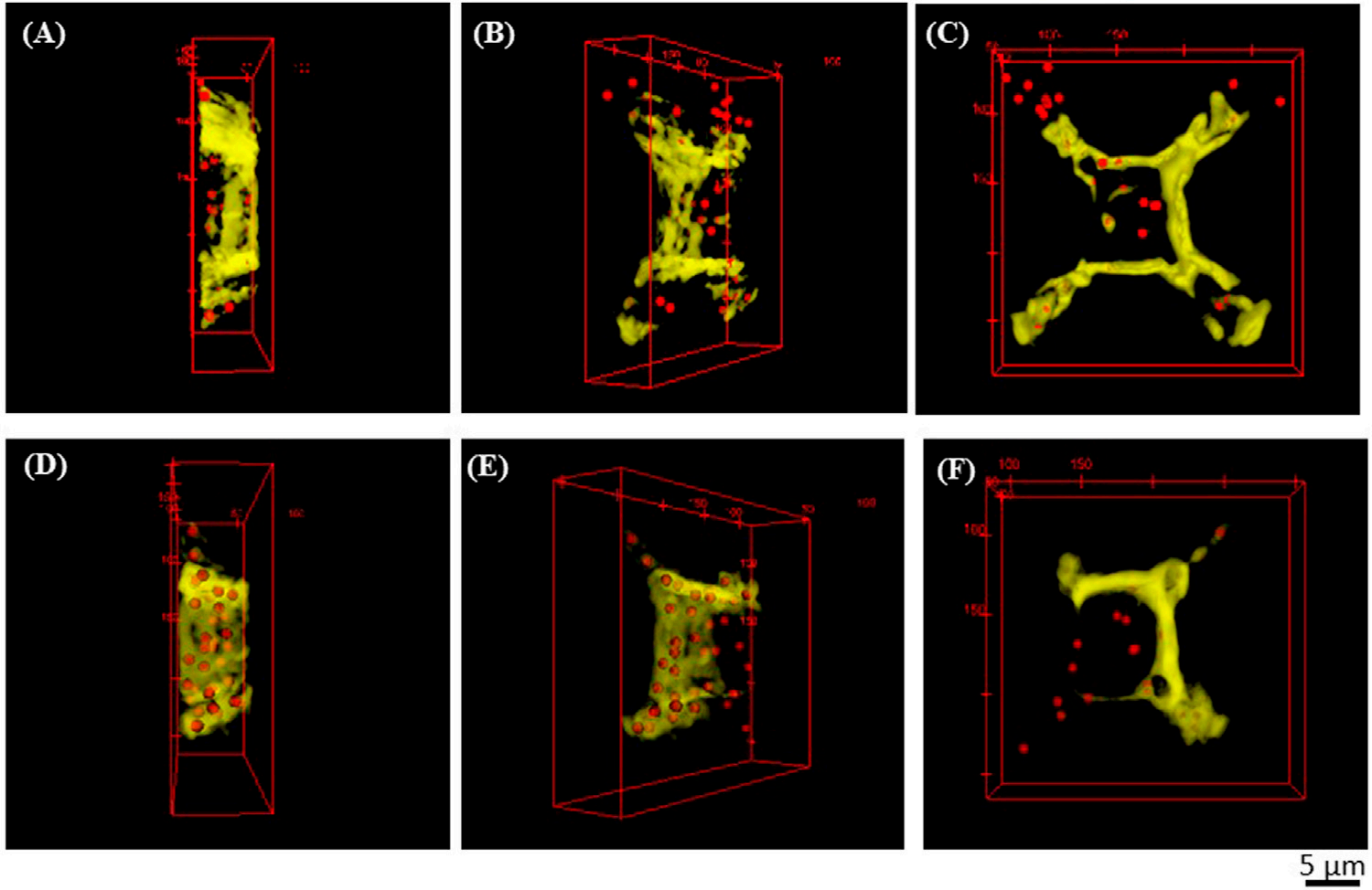
**(A,D)** Lateral views, **(B,E)** Tilted views and **(C,F)** Top views obtained by enhanced dark field microscopy of a microcage (as the one displayed in Figure 4J). The microcage’s walls were colored yellow and the centers of MNPs and MNPs aggregates were colored in red (false colors). From **(A)** to **(D)**, from **(B)** to **(E)** and from **(C)** to **(F)** the microcage was rotated with 180° on the vertical axis.

To evaluate the presence of MNPs in the resulting micro‐cages, we investigated the microcage‐like structures using enhanced dark field microscopy. Two sets of images were acquired, as following: one set of images was the Z‐scanning fluorescent images structures and the second set was the Z‐scanned images in “white light”, the later set of images related to the scattered radiation by the MNPs (as it was explained in the previous section). These two sets of experimental images were processed for obtaining 3D reconstructed images that combine the two types of contents: the structures of the microcages and the MNPs located inside them (Figures 5, 6). Given the differences in light scattering coming from the sample composition, the presence of the MNPs and MNPs aggregates could be emphasized at a depth of 50 µm inside the volume of the structures (Figures 5, 6, red dots). In the experimental images, the photopolymer autofluorescence is in the green domain; in Figures 5, 6, we used false colors: yellow for the IP‐L780 photopolymer, while the cores of MNPs and MNPs aggregates were represented in red. In Figure 5, the images were captured at various focusing depths on the micro‐cage, as following: focus on the top of the microcage (Figure 5A), followed by a stack of images from the top of the microcage‐like structure towards the bottom, which were obtained by incremental focusing depths inside the microcage structure (Figures 5B–F).

More relevant images are displayed in Figure 6. The lateral views (Figures 6A, D), the tilted views (Figures 6B, E) and the top views (Figures 6C, F) were obtained by 3D reconstructions from experimental Z‐stacks of a microcage‐like structure (like the one displayed in Figure 4J), which was rotated at different angles. Therefore, in Figures 6A–F, the microcage‐like structure was rotated 180° on the vertical axis. These images provided additional and undoubtable evidence that the magnetic nanoparticles were relatively homogeneously dispersed within the entire volume of the microcage‐like structures. The movie with the MNPs embedded in the polymer matrix inside the microcage‐like structure is available in the Supplementary Material.

**FIGURE 7:**
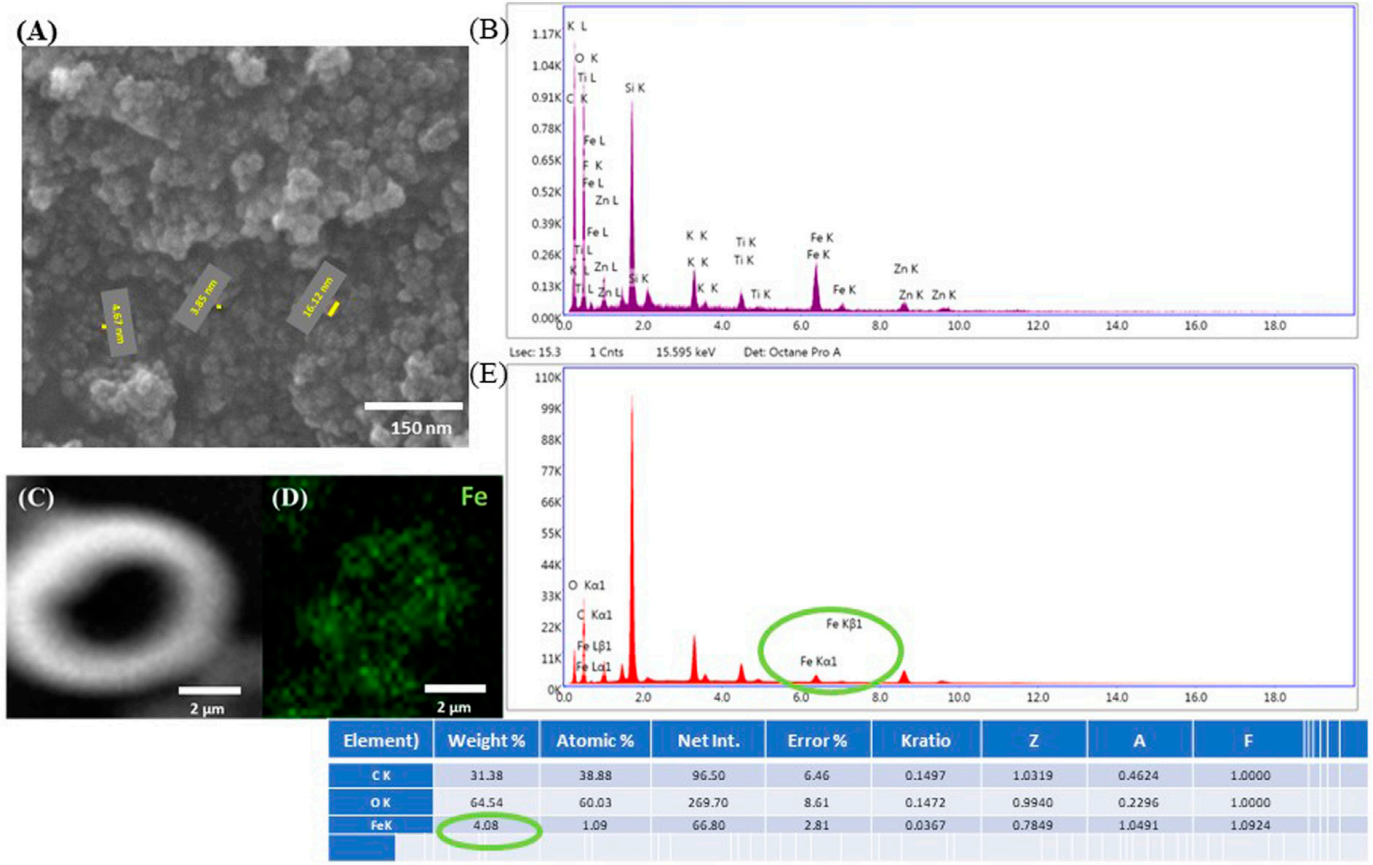
**(A)** Scanning electron micrographs of native IP-L780/MNPs composite. The yellow segments indicate the diameter of nanoparticles, with the numerical values specified next to each segment (diameter size between 3 and 17 nm). **(B)** EDX spectrum of unpolymerized IP-L780/MNPs composite, with MNPs concentration of 4 mg/mL. The spectra show the presence of Fe, indicative of the fact that the nanostructures observed in **(A)** are magnetic nanoparticles. **(C)** Scanning electron micrograph of a test structure made of a vertical microtube (as shown in Figure 2); **(D)** EDX mapping of Fe in the structure shown at **(C)**; **(E)** EDX spectrum and table with compositional analysis of the structure shown at **(C)**.

Additionally, energy dispersive X‐Ray spectroscopy (EDX) was employed to confirm the elemental composition of the submicrometric inclusions identified in the microcage structure. In order to prove the principle of the technique, both SEM and EDX were employed on the polymerized IP‐L780/MNPs composite used to laser‐imprint the microcage‐like constructions (Figure 7). Here, one can clearly see the nanometric morphology of the MNPs in the un‐polymerized IP‐L780/MNPs composite, resulting in flower‐like aggregated structures (Figure 7A). The EDX spectrum confirms the presence of superparamagnetic nanoparticles, showing specific peaks for Fe element (Figure 7B). Figure 7 also provide evidence on the fact that the magnetic nanoparticles preserved their homogenous distribution even after the photopolymerization process. Thus, Figure 7C shows a SEM micrograph of laser‐imprinted microtube made of IP‐L780/MNPs composite, Figure 7D shows results on EDX mapping on the microtube from C), indicating the presence of Fe in the imprinted structure. Figure 7E shows the EDX spectrum and table with compositional analysis indicating the presence of Fe within the microtube (thus within the polymerized IP‐L780/MNPS composite) and the table gives numerical values on the elemental composition of the microtube from Figure 7C.

**FIGURE 8:**
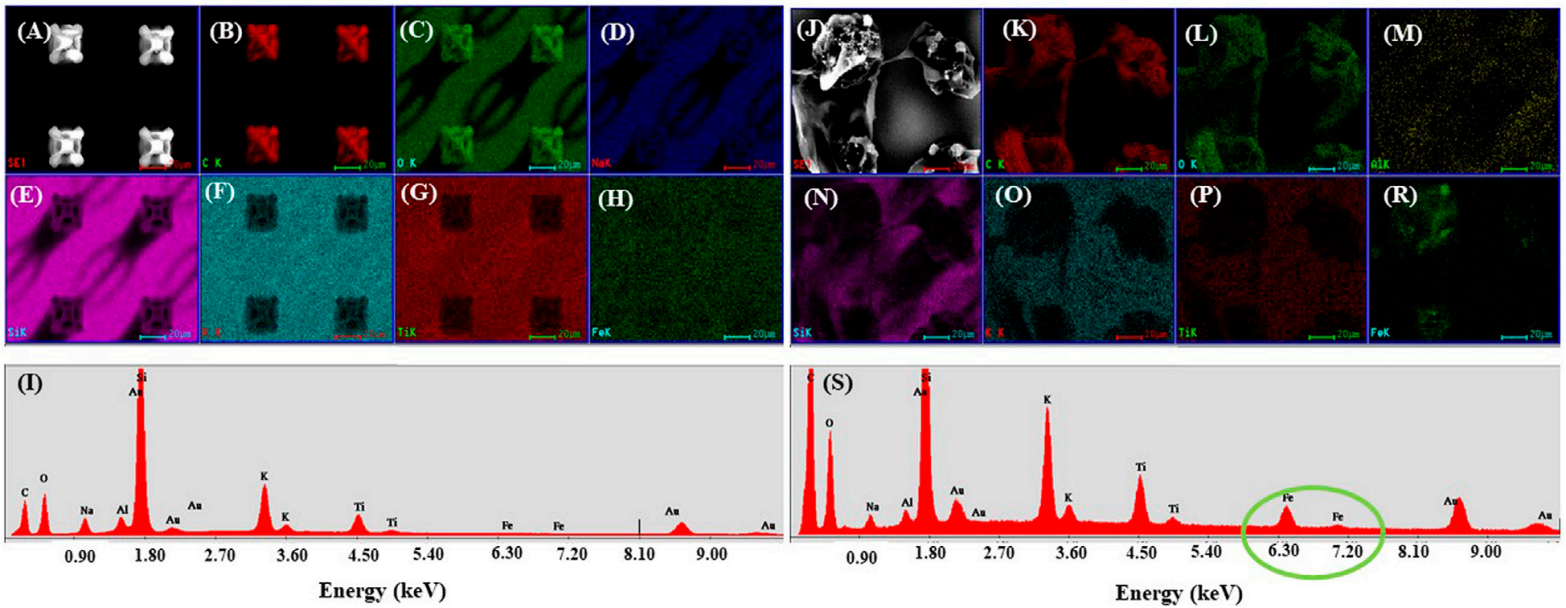
Scanning electron micrograph of microcages made by LDW via TPP of IP-L780 **(A)** and IP-L780/MNPs composite **(B)**; EDX mapping of microcages arrays made of IP-L780 **(B–H)** and IP-L780/MNPs composite **(K–R)**. The maps show the spatial distribution, for left panel, of the following elements: C, O, Na, Si, K, Ti, Fe; **(I,S)** EDX spectra of microcages structures from **(A,J)** respectively, showing the presence of Fe only in the IP-L780/MNPs samples, indicative of the fact that the microcages made by LDW via TPP of IP-L780/MNPs contain magnetic nanoparticles.

Then, EDX mapping was employed to demonstrate the presence of MNPs in the laser‐imprinted microcage‐like structures (Figure 8). Because the depth penetration of the electrons in samples at the employed energy is below 10 μm, we have used composite constructs with altered morphology (structures which exploded under the laser beam, due to tension in the composite material at the interface between MNPs‐photopolymer). This enabled us to “see” deeply into the 3D structures and to identify the elemental composition of the microcage‐like structures (Figure 8I as compared to Figure 8S).

In order to prove the ability of the IPL‐780 photopolymer/MNPs microcage‐like structures to entrap osteoblast‐like cells through static magnetic field (SMF) activation, the following experimental conditions were taken into consideration: 1) microcage‐like structures made of IP‐L780 photopolymer alone in absence of SMF (IP‐L780), 2) iPL+SMF: microcage‐like structures made of IP‐L780 alone in presence of SMF (IP‐L780+SMF), 3) microcage‐like structures made of IP‐L780/MNPs composite without SMF (IP‐L780/MNPs), 4) microcage‐like structures made of IP‐L780/MNPs composite with SMF (IP‐L780/MNPs+SMF).

Before presenting the *in vitro* results, one must emphasize the fact that the biological processes cannot be controlled in a 100% manner. Instead, for *in vitro* studies, the scientific output is provided by the trends observed within the experiments. In our case, the main population of cells was entrapped in the microcage‐like structures in case of IP‐L780/MNPs with SMF; indeed, there were also some cells on the exterior of the microcages, because IP‐L780 photopolymer is biocompatible and possesses certain adherence properties in relation to the seeded cells. The purpose of the study was not to use cell repellent materials, but to provide a biomimetic environment for the cells to grow at specific places of the structures. However, we agree that the system can be improved by inscribing the microcages onto a cell repellent surface, in order to reduce the number of cells at the bottom of the microcage‐like structures, which will be the subject of our studies in the nearest future. In the present work, we used coverslip glass as substrate for the laser‐imprinted microcage‐like structures, which is an adherent surface for cells.

**FIGURE 9:**
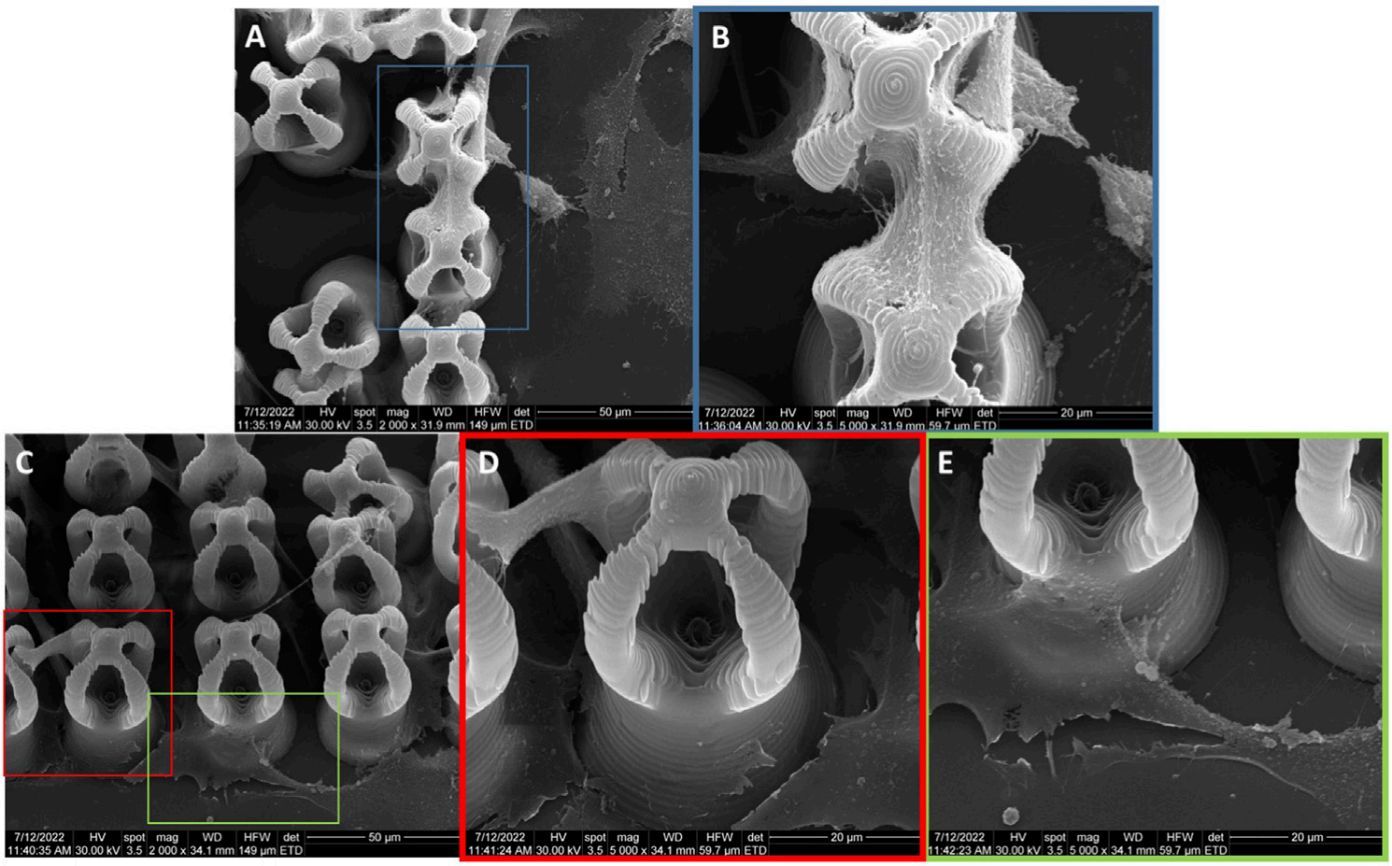
General overview and details of MG-63 osteoblast-like cells morphology on microcage-like structures made of IP-L780 alone, without magnetic field stimulation; blue, red and green squares limit an area which was evaluated in detail, at higher magnification; **(A,B)** -top view and **(C–E)**- titled 45^o^ view.

Scanning electron micrographs indicate that MG‐63 osteoblast‐like cells cultured on microcage‐like structures made of IP‐L780 photopolymer alone, with no magnetic nanoparticles embedded in their composition and in absence of any magnetic field stimulation, had a polygonal morphology; most of the cells were situated at the bottom of the structures and at the exterior of the top part, covering the microcage‐like structure (Figure 9). The attachment points were not well‐defined. Details of the attachment points (Figures 9D, E) emphasize the rough steep edges of the osteoblasts and their inability to form strong attachment bonds with the substrate.

The scope of this study was to design, fabricate and characterize microcage‐like structures in what concerns their ability to entrap osteoblast‐like cells, for guiding the cells adhesion to very specific places within complex architectures. The work intends to demonstrate a proof of concept regarding a selective cellular attachment in architecturally‐controlled 3D environments and targets further tissue engineering applications. Until present, the main method to investigate cell‐cell and cell‐structures interactions is considered to be Scanning Electron Microscopy (SEM). This imaging technique has been broadly used to emphasize the ability of cells to adhere and migrate in 3D structures (Roy et al., 2002; Pijuan et al., 2019; Vanslembrouck et al., 2022), as well as the ability of 3D structures to entrap cells (Sharaf et al., 2022; Larramendy et al., 2019; He et al., 2022; Spagnolo et al., 2015; Subbiah et al., 2020). The efficiency of SEM relies on the generally accepted fact that it provides a sufficiently high resolution to allow the detailed observation of the cells morphology and of the attachment points of cells seeded on various 3D structures, as well as to analyze the adherence of cells on 3D scaffolds (Diban et al., 2014; Tian et al., 2019; Polak et al., 2023). According to the above‐mentioned reasons, for our study, Scanning Electron Microscopy was the best method to emphasize the fact that osteoblast cells were able to adhere to the microcage‐like structures, to evaluate the cells morphology in contact with the structures and to determine, with high spatial accuracy, the position of the cells in relation to the different parts of the microstructures. Another technique that is often used to image cells is fluorescent microscopy. However, due to the experimental conditions of our study, namely, the auto‐fluorescence of the microcage‐like structures (more precisely, of IP‐L780 photopolymer), it was impossible to emphasize the presence of the cells on the structures using this imaging technique. Supplementary Figure S1 illustrates the impossibility to distinguish the cells from the microcage‐like structures, despite using various fluorescent markers for the cells. The strong autofluorescence in green, blue and red of the microcage‐like structures hindered the visualization of the seeded cells. In Supplementary Figure S1, the cells were marked with acridine orange (emits in green), but it should be noted that other markers (i.e., that emit in other colors such as blue or red) would not be helpful in distinguish the cells from the structures either, because, as we already mentioned, the structures showed a strong autofluorescence not only in green, but also blue and red.

The viability of the cells cultured on the microcage‐like structures is proved by the star‐shaped/polygonal morphology, with strong adherence points. These features are generally recognized as specific morphological characteristic that indicate that the cells are viable; moreover, it is widely accepted that the polygonal morphology is a normal morphology for viable osteoblast cells (Rutkovskiy et al., 2016; Paun et al, 2018a; Paun et al, 2018b; Qian et al., 2021). In contrast, a non‐viable cell would be round‐shaped, which was not observed in our experimental findings (Balvan et al., 2015).

In our study, the microscopic investigation of the cells morphology following their interaction with the structures revealed that the cells showed a polygonal shape, which, as emphasized by previously published works (Rabel et al., 2020; Paun et al, 2018a; Paun et al, 2018b), is a normal shape of a viable cell. These findings provide evidence that the microcage‐like structures made by IP‐L780/MNPs superparamagnetic composite offer a biocompatible environment for osteoblast‐like cells.

As previously stated, the auto‐fluorescent IP‐L780/MNP structures are not suitable for fluorescence microscopy. Fluorescence microscopy could be another technique to employ in order to prove the purpose of the study, however, due to the auto‐fluorescence of the microcage‐like structures (specifically of IP‐L780 photopolymer) it is impossible to emphasize the presence of the cells. Please, find the example illustrated in the figure below, that clearly proves the inutility of fluorescent marking of cells seeded on microcage‐like structures. The strong autofluorescence in green of the microcages hinders the visualization of the seeded cells (marked with acridine orange). To be noted that other markers will act the same, because the microcages have a strong autofluorescence in blue and also red.

**FIGURE 10:**
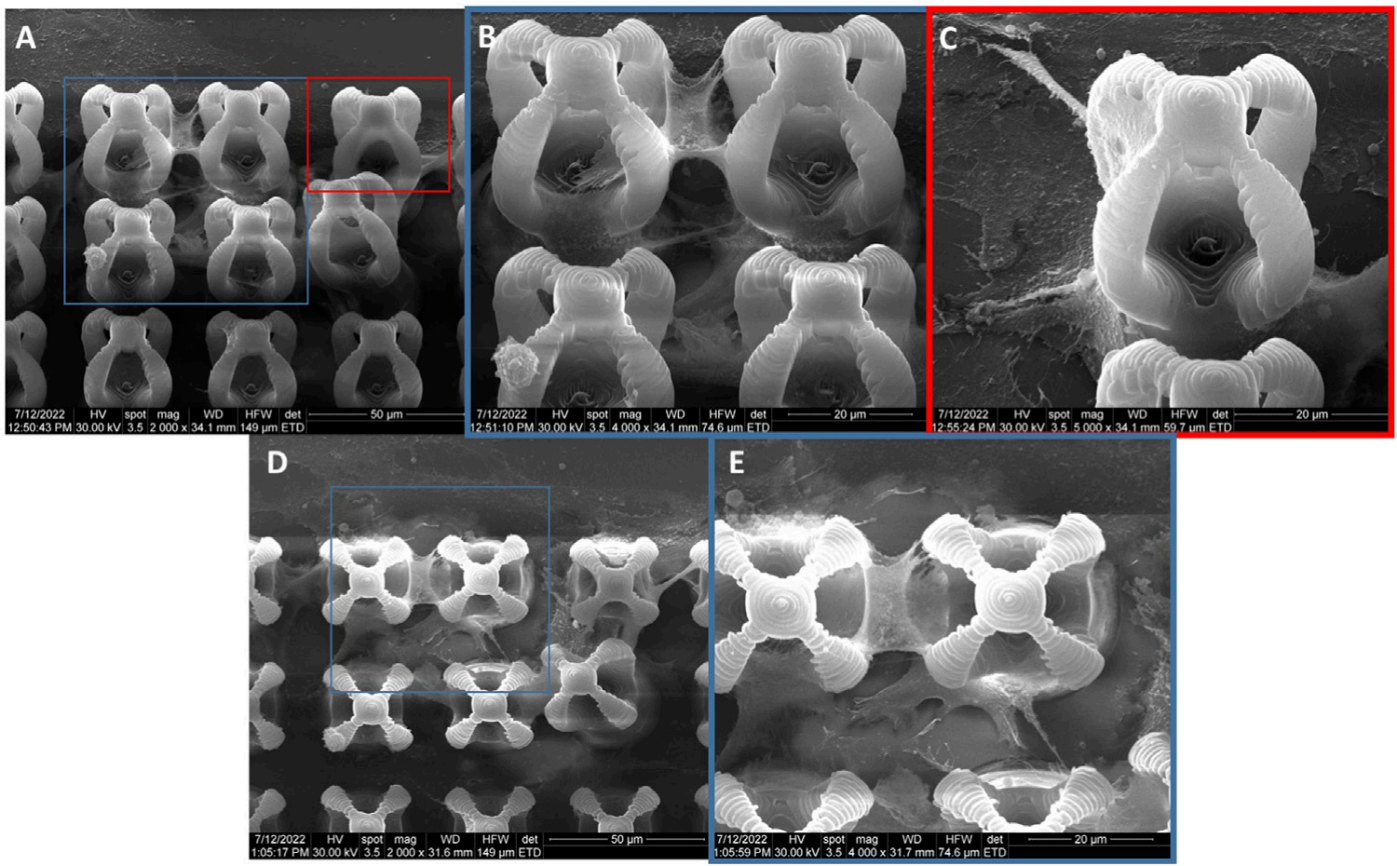
General overview and details of MG-63 osteoblast-like cells morphology on microcage-like structures made of IP-L780 alone, with magnetic field stimulation; blue and red squares limit an area which was evaluated in detail, at higher magnification; **(A–C)**- tilted 45^o^ view and **(D,E)**- top view.

The cells seeded on microcage‐like structures made of IP‐L780 photopolymer alone and that were stimulated by external static magnetic field were also mostly attached at the bottom and on the outer parts of the microcage‐like structures (Figure 10). Here, the attachment points were better emphasized, and smoother compared to the non‐stimulated structures (Figures 10B, E), suggesting that the external magnetic field on its own aids in the improvement of cells‐biomaterial interactions (Filippi et al., 2022).

**FIGURE 11:**
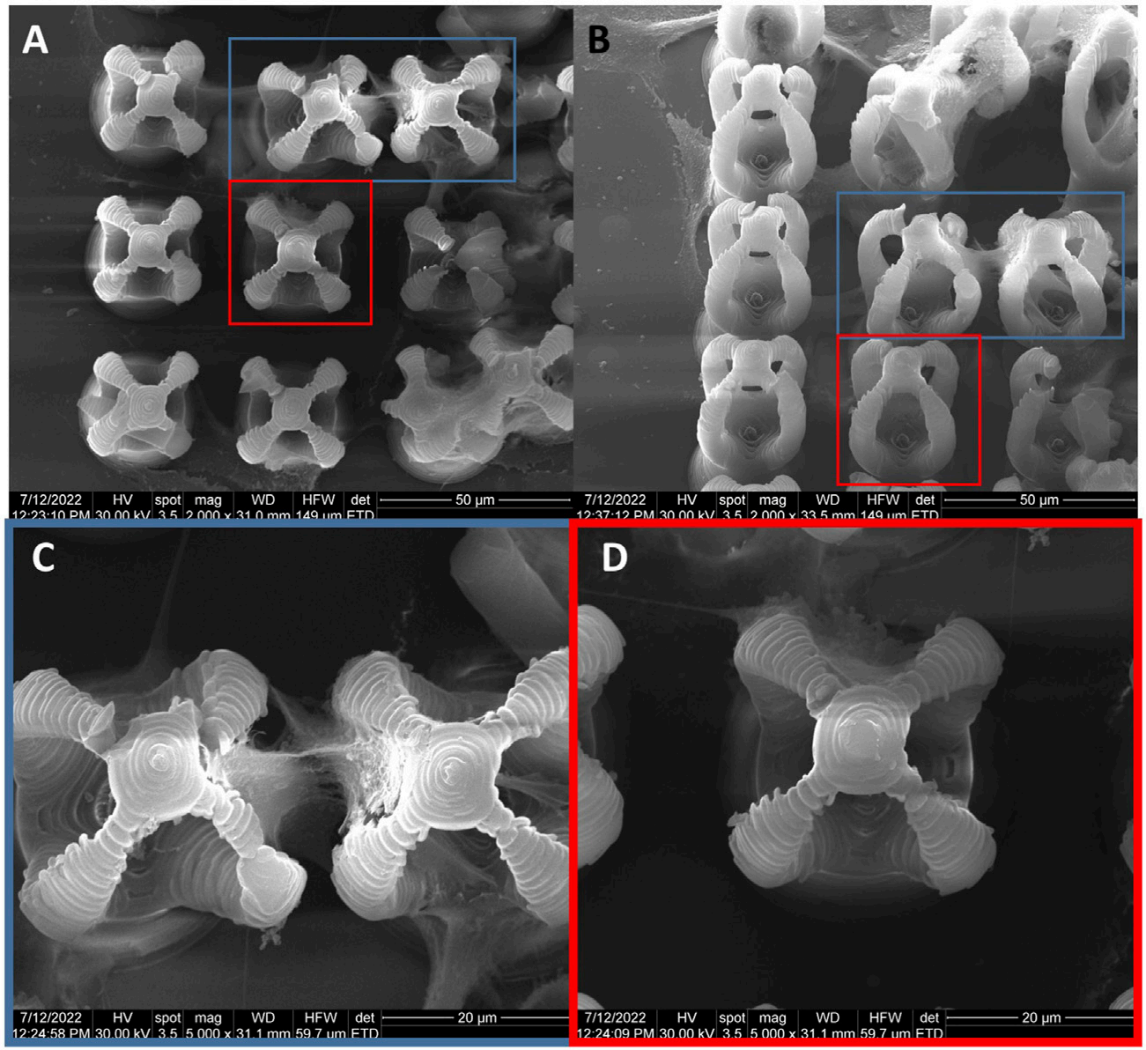
General overview of MG-63 osteoblast-like cells morphology on microcage-like structures made of IP-L780/MNPs composite, without magnetic field stimulation; blue and red squares limit an area which was evaluated in detail, at higher magnification; **(A)**, **(C,D)**- top view and **(B)**- tilted 45^o^ view.

The cells seeded on microcage‐like structures made of IP‐L780/MNPs composite and in absence of magnetic field stimulation (Figure 11) showcased a normal polygonal morphology of the osteoblast‐like cells, which were situated at the bottom, in the exterior as well as in the inner parts of the microcage‐like structures. The nanopatterned surface of the structures given by the presence of MNPs embedded in the IP‐L780 photopolymer enabled many attachment points for the osteoblasts, whose filopodia extended smoothly in order to spread onto the complex architecture of the microcages.

**FIGURE 12:**
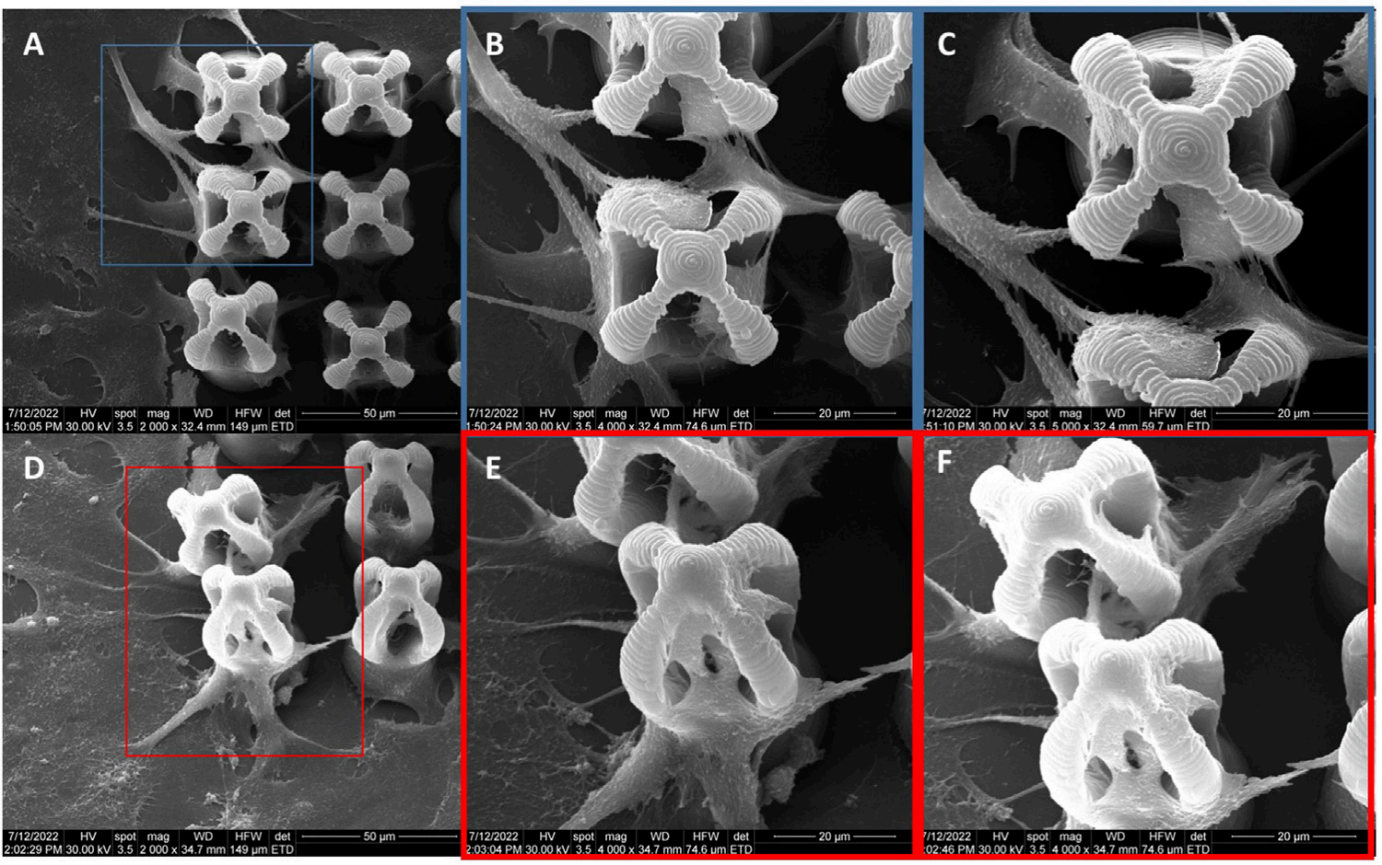
General overview of MG-63 osteoblast-like cells morphology on microcage-like structures made of IP-L780/MNPs composite, with external static magnetic field stimulation; blue and red squares limit an area which was evaluated in detail, at higher magnification; **(A–C)**- top view and **(D–F)**- tilted 45^o^ view.

The osteoblast cells that adhered on microcage‐like structures made of IP‐L780/MNPs and that were stimulated with static magnetic fields showcased an enhanced elongated star‐like morphology compared to non‐stimulated structures. The cells exhibited long filopodia which allowed them to act like bridges between microcages, as well as in‐between the bottom and the top part of the structures. In this case, the scanning electron microscopy images clearly indicated that most of the cells were situated inside the microcage‐like structures. There were very few cells situated on the exterior part of the microcages, most of them acting like connections between the bottom pillar of the structure and the top microcage part (Figure 12).

**FIGURE 13:**
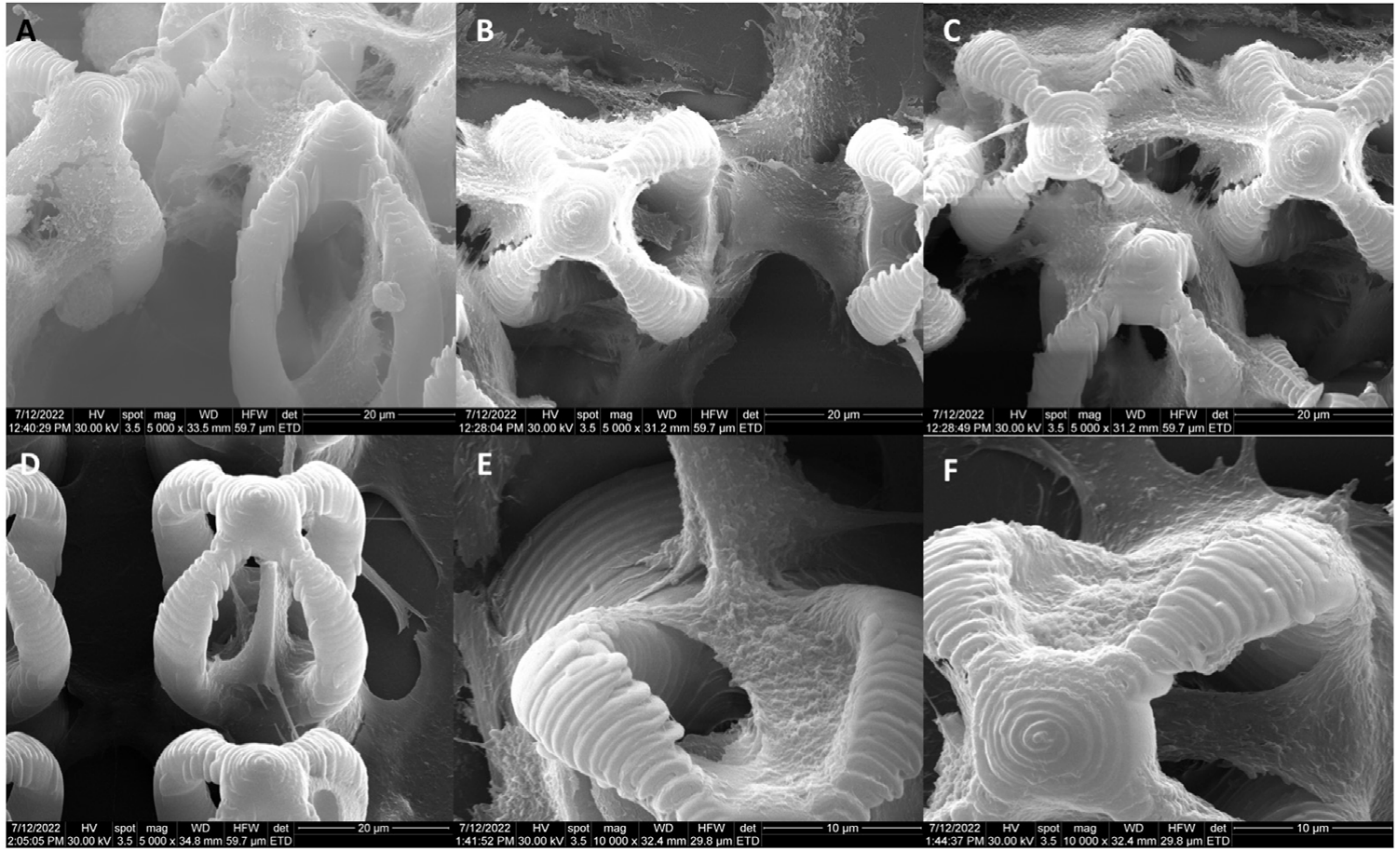
Detailed view of MG-63 osteoblast-like cells morphology on microcage-like structures made of IP-L780MNPs composite: **(A–C)** without external static magnetic field stimulation, **(D–F)** with external static magnetic field stimulation; tilted 45^o^ view.

Although there were some cells trying to enter inside the inner part of the microcage‐like structures in the absence of a magnetic field (Figures 13A–C), most of them only covered the exterior of the structures. In this case, the osteoblasts exhibited fragile attachment points, the filopodia being very thin, many of them being deteriorated due to the repetitive washing steps in the harsh dehydrating protocol for scanning electron microscopy preparation. It has been previously shown that magnetic field stimulation has effects on the cell’s cytoskeleton organization (Wu et al., 2018). Filopodia are direct extensions of the actin filaments and exhibit an active role in the cells‐biomaterial interactions such as attachment to the substrate and motility. In the case of static magnetic field stimulated structures made of IP‐L780/MNPs composite, the osteoblast cells colonize the interior of the microcage‐like structures, being attracted by the top inner part of the structure (Figures 13D–F). The cells showed a star‐shaped morphology and the attachment points looked strong, as they were thick and well‐defined on the structures.

**FIGURE 14:**
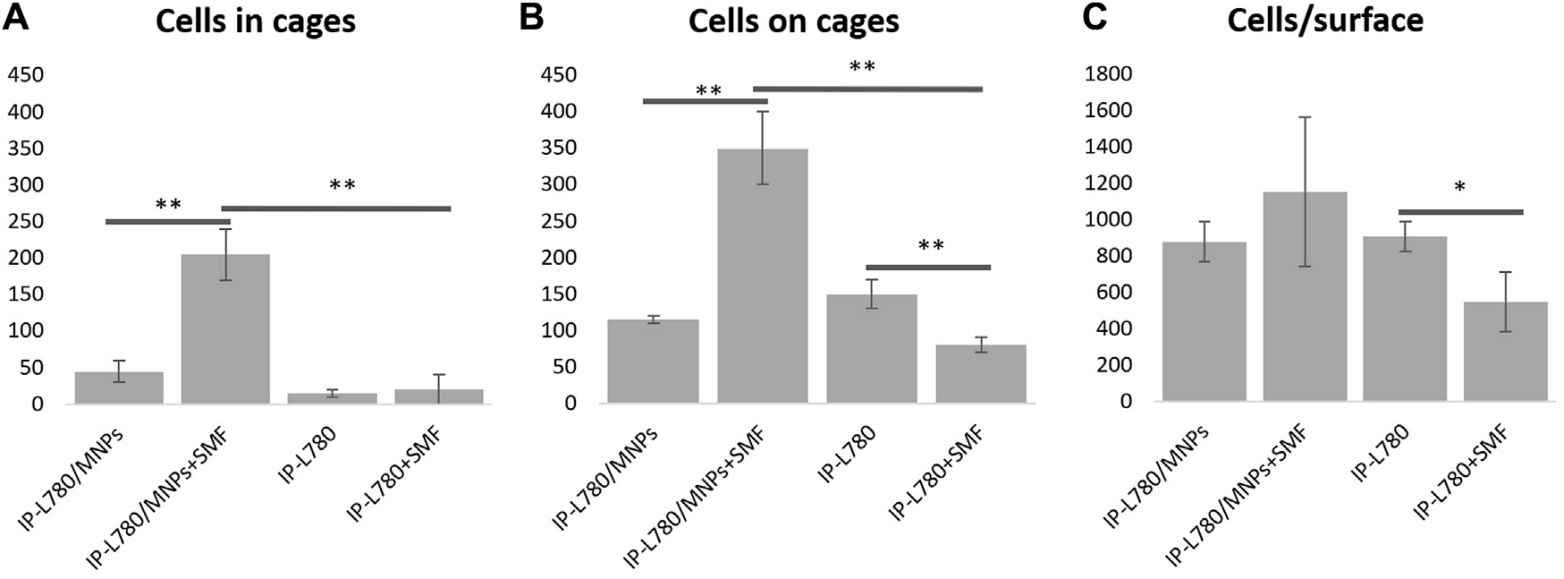
Cell scoring in microcage-like structures made of IP-L780 photopolymer alone or IP-L780/MNPs composite with/without external magnetic field stimulation: **(A)** number of cells inside the microcage-like structures; **(B)** number of cells on the exterior of the microcage-like structures; **(C)** total number of cells relative to the whole microstructured surface; where IP-L780/MNPs- IP-L780 microcage-like structures containing MNPs without magnetic field stimulation (SMF), IP-L780/MNPs+SMF - IP-L780 microcage-like structures containing MNPs with SMF, IP-L780- IP-L780 microcage-like structures without MNPs and respectively without SMF, IP-L780+SMF- IP-L780 microcage-like structures without MNPs with SMF; *p < 0.05, **p <= 0.01 and respectively ***p <= 0.001.

In order to have a numerical evaluation of the developed microcage‐like structures made of IP‐L780/MNPs composite ability to entrap the cells under external magnetic field activation, a cell counting was performed based on the scanning electron microscopy images obtained for MG‐63 osteoblast‐like cells cultured on the samples with/without magnetic field stimulation (Figure 14). Thus, for each of the samples considered in the proposed experimental design were scored cells inside the cages (completely situated in the interior, but as well cells that were partly spreading outside the core‐ex. cells connecting the interior of two microcages). Separately, cells situated on the exterior part of the microcage‐like structure were counted (on the “tentacles”, as well as the leg stand). Lastly, the whole number of cells situated in the microcage area were counted, here being included the cells at the bottom of the microcage‐like structures or in‐between. This was done in order to have an estimation of the cell occupation in respect to the polymer‐based micro‐patterned material, as well the proximal glass substrate support. For this, the area of each sample was calculated based on the scanning electron microscopy measurements and the total number of cells scored for the respective sample was divided by the calculated area (in mm^2^).

Specifically, for IP‐L780 alone we counted 15 ± 5 cells inside the cages, while 150 ± 20 cells were on the exterior of the cages, with a total cells/surface ratio of 907 ± 82.45. In case of actuated IP‐L780 (IP‐L780+SMF), we counted 20 ± 20 cells inside the cages and 80 ± 10 cells on the exterior of the cages, with a total cells/surface ratio of 549.7 ± 164.9. In the case of the non‐actuated composite IP‐L780/MNPs we counted 45 ± 15 cells inside the cages and respectively 115 ± 5 cells on the exterior of the cages, with a total cells/surface ratio of 879.5 ± 109.9. For magnetically actuated composite samples IP‐L780/MNPs + SMF we counted 205 ± 35 cells in cages and respectively 350 ± 50 cells on the exterior of the cages, with a total cells/surface ratio of 1154.36 ± 412.27. This highlighted the fact that the magnetic actuation of IP‐L780/MNPs composite enables cells entrapment in the interior of the cages, as well as an enhancement of total cell number (cell viability).

Irrespective of the presence of MNPs or the SMF, the cell occupancy was similar for all samples (NS differences), apart from the microcage‐like structure made of IP‐L780 photopolymer alone with magnetic field stimulation, where a reduction in total cell population was remarked (*p* < 0.05, IP‐L780+SMF compared to IP‐L780). This shows that in the absence of MNPs, the microcage‐like structures made of IP‐L780 photopolymer alone can support the attachment and growth of osteoblast‐like cells, but when a static magnetic field is applied, cell occupancy is slightly inhibited. Although magnetic field stimulation has been previously proved to enhance the growth of MG‐63 osteoblast‐like cells (Paun et al., 2018b; Li et al., 2019), this small noticeable inhibitory effect in case of the microcage‐like structures made of IP‐L780 photopolymer alone must be explained by the complex architecture of the sample.

Nevertheless, external static magnetic field stimulation had a positive effect on the cell entrapment in microcage‐like structures made of IP‐L780/MNPs composite, showing a significant increase of cell number with about 4.5 folds (*p* < 0.01) compared to non‐stimulated samples (Figure 14A). Moreover, the addition of MNPs in the microcage‐like structures demonstrated a positive effect in the cell entrapment ability, in samples with SMF, as IP‐L780/MNPs+SMF showed a 10.25 folds increase in cell cage entrapment, compared to IP‐L780+SMF (*p* < 0.01). The addition of MNPs alone in the samples did show a slightly small improvement in microcage entrapment ability, however the effect was not statistically significant (IP‐L780/MNPs compared to IP‐L780 alone, NS).

The relatively high standard deviation regarding the cell entrapment efficiency (±5.11) could, at a first look, indicate a considerable variability. Although 5% might be a high error in chemistry and physics, it is broadly accepted in biology, due to the high variability induced by the living biological material (Lacombe et al., 2020; Bacakova et al., 2022; He et al., 2022). Moreover, our statements and conclusions are based on statistical analysis of the data, which was performed by Studet *t*‐test (described in Materials and methods section), which is a common statistical method applied in biomaterials characterization (Lacombe et al., 2020; Bacakova et al., 2022; He et al., 2022).

A significant population of cells was attracted by the exterior of the microcage‐like structures, which offered cell growth support. Thus, the external magnetic field‐stimulated microcage‐like structures made of IP‐L780/MNPs composite showed a much higher and significant increase in the ability to support cell attachment, compared to all other structures (*p* < 0.01). Similar as in the total number of scored cells, microcage‐like structures made of IP‐L780 photopolymer alone showed an inhibition of osteoblast‐like cell attachment on the exterior of the microcage‐like structures when SMF activation was done (1.87 folds more cells in IP‐L780 compared to IP‐L780+SMF, *p* < 0.01). Thus, it seems that the addition of MNPs in the microcage‐like structures has a significant positive impact in the cell attachment (*p* < 0.01 for IP‐L780/MNPs+SMF compared to IP‐L780+SMF), as well as microcage cell entrapment (*p* < 0.01 for IP‐L780/MNPs+SMF compared to IP‐L780+SMF). These numerical results strongly sustain the morphological observations concerning the use of magnetic actuation on microcage‐like structures made of IP‐L780/MNPs composite for osteoblast‐like cells restrained growth in tissue engineering applications.

Furthermore, the use of SEM in this study provided crucial insights into the morphology and distribution of cells on the microcage structures composed of the IP‐L780/MNPs composite. SEM allowed us to precisely observe the shape of osteoblastic cells and their locations both at the base and inside these microcages. SEM has been previously employed to analyze the distribution of cells on a surface or within a three‐dimensional structure (Iandolo et al., 2019); this technique enabled researchers to determine the cell density and their spatial distribution. SEM was previously used to quantify the surface texture of cells, involving evaluating surface roughness or the presence of distinctive cellular features like protrusions or microvilli (Accardo et al., 2017; Domínguez-Bajo et al., 2020; Jing et al., 2021). In the present study, SEM investigations of cells seeded on glass slides (negative controls) enables us to evidence the polygonal, star‐shaped morphology with pronounced protrusions (attachment points) of the osteoblast‐like cells (Supplementary Figure S2).

Additionally, through SEM, we were able to analyze the intricate details of the nanopattern resulting from the incorporation of MNPs in the IP‐L780 photopolymer. This aspect was particularly significant as it furnished information about multiple attachment sites for osteoblastic cells on these structures. SEM was previously used to quantify the surface texture of cells, involving evaluating surface roughness or the presence of distinctive features like protrusions or microvilli (Accardo et al., 2017; Domínguez-Bajo et al., 2020; Jing et al., 2021). Scanning electron microscopy provides a sufficiently high resolution to be able to observe the details of osteoblast cells attachment points and was used before to analyze the adherence of different cells onto scaffolds (Diban et al., 2014; Tian et al., 2019; Polak et al., 2023).

**FIGURE 15:**
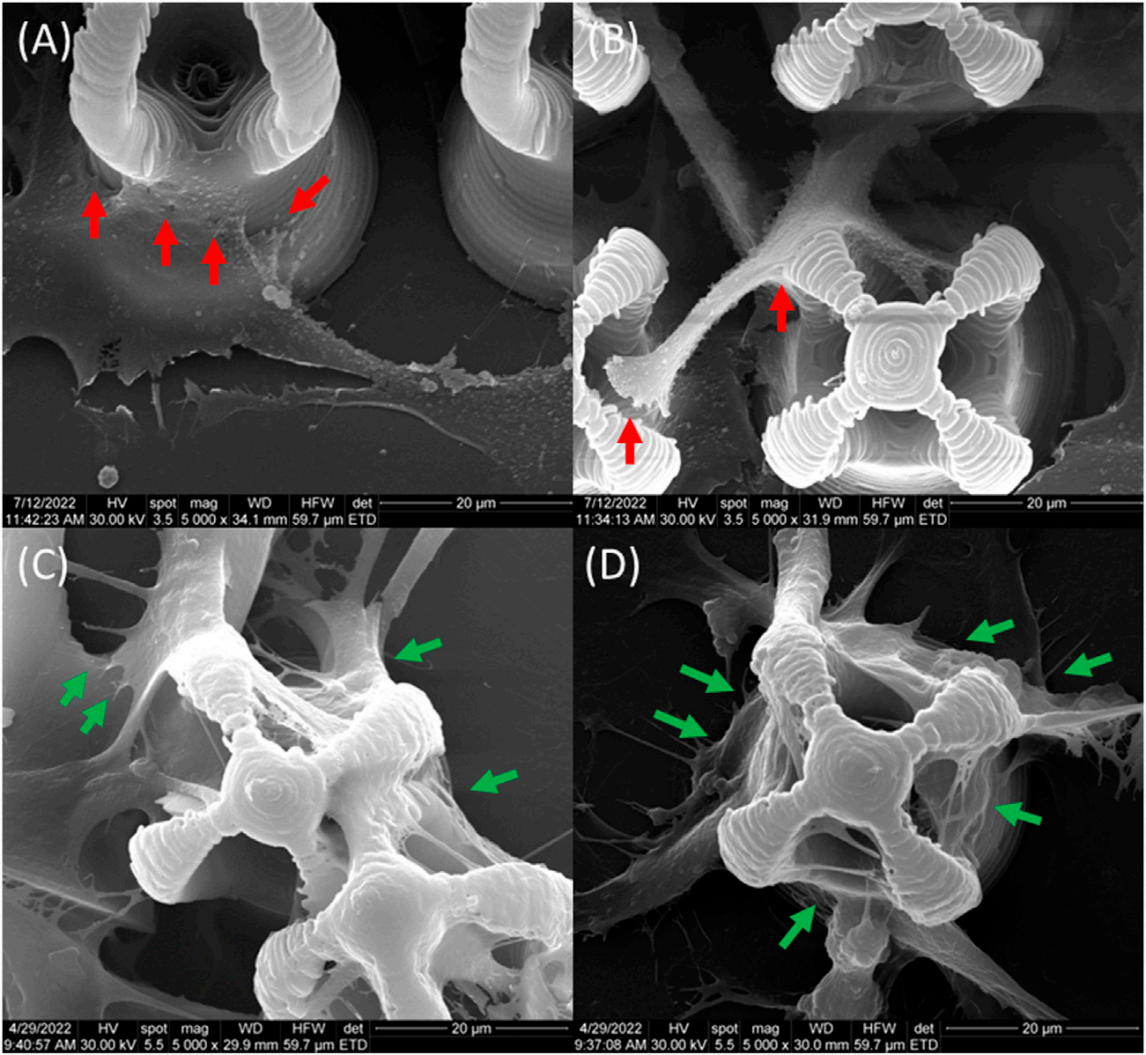
Morphological features of MG-63 osteoblast like cells cultured on **(A,B)** IP-L780 non-actuated and respectively on **(C,D)** magnetically actuated IP-L780/MNPs; red arrows-small steep attachment points, green arrows-long, strong well-anchored attachment points.

Based on the above considerations, in our study, SEM imaging was employed to monitor the cellular attachment within the microcage‐like structures, due to the ability to observe the morphology of the cells cultured on 3D structures at submicron resolution, as demonstrated by the successful use of SEM technique in numerous similar *in vitro* studies (Larramendy et al., 2019; Sharaf et al., 2022). Hence, we were able to highlight the morphological features that characterize the cellular behavior i.e., attachment when seeded on the microstructures and the differences between microcage‐like structures made by IP‐L780 photopolymer alone and the structures made of IP‐L780/MNPs composite and actuated by Static Magnetic Fields. Figure 15 provides a detailed analysis of the morphological features of MG‐63 osteoblast like cells cultured on IP‐L780 non‐actuated and respectively on magnetically actuated IP‐L780/MNPs microcage‐like structures. The experimental findings show that the cell seeded on microcage‐like structures made from IP‐L780/MNPs composite and actuated in SMF possess strong well‐anchored attachment points (Figures 15C, D), in contrast with the weak attachment points provided by the structures made from IP‐L780 alone (Figures 15A, B).

## 4 Discussion

Over time, 3D printing has been extensively employed in the advancement of elaborate micrometric architectures with complex functions. Nature itself has served as inspiration in the development of such models, for example, leaf hairs in Salvinia molesta having the ability to retain air when exposed to water abundant environments (Omar et al., 2015). This has been successfully accomplished through the employment of laser direct writing technologies. Moreover, LDW technologies have been translated in the development of 4D external stimuli‐actuated micro‐machines (Jin et al., 2020), completing the mission to obtain micro‐robot structures able to fulfill different biological tasks.

Here, we have proposed a complex microcage‐like structure which can be activated through external magnetic field stimulation into the entrapment osteoblast cells for tissue engineering applications. The incorporation of MNPs into the microcage‐like photopolymeric structure was done in order to induce a magnetic property to the structure for obtaining a magnetically‐active device. Due to their superparamagnetic behavior, the iron oxide nanoparticles were magnetically‐activated only in the presence of an external magnetic field (Wahajuddin, 2012). Here, polyethylene glycol‐encapsulated iron oxide nanoparticles (Popescu et al., 2020) were used to facilitate the incorporation of the inorganic phase into the organic photopolymer and to improve the dispersion of the MNPs in the liquid IP‐L780 photopolymer. The homogenous localization of the MNPs inside microcage‐like structure could be clearly observed in 3D reconstructed images recorded by enhanced dark field microscopy, using a method that has been recently used to characterize complex 3D composite structures (Paun et al, 2020b).

Unlike barrier containment structures, which presume an additional step to “close” the barrier following the entrapment of the cells inside the cages, in the case of IP‐L780/MNPs microcage‐like structures, the osteoblasts attachment and growth was guided through magnetic field stimulation. The advantage is that there were no additional crosslinking agents needed (Veernala et al., 2021), nor supplementary fabrication of stacking layers must be applied (Larramendy et al., 2019), which can be harsh and damaging for the internalized cells. The microcage‐like structures offer a biocompatible substrate for osteoblast‐like cells to adhere and to grow. This was emphasized through microscopic investigation of the cells morphology following interaction with the structures. Our observations concluded that the cells show a normal aspect, as emphasized by previous studies (Rabel et al., 2020; Paun et al, 2018a; Paun et al, 2018b).

The biocompatibility of IP‐L780/MNPs superparamagnetic composite material used for structures’ fabrication as well as cells viability were evaluated by us in our recent works. Namely, we have used the superparamagnetic IP‐L780/MNPs composite to obtain biomimetic structures with different architectures, for bone tissue engineering (Paun et al, 2018a; Paun et al., 2019). These microstructures were cultured with MG‐63 osteoblast‐like cells (which is the same cell line as the used in the present study) (Paun et al., 2018b; Paun et al., 2019). In those studies, we evaluated the cells viability on structures made of IP‐L780/MNPs superparamagnetic composite using a tetrazolium‐salt MTS assay. We found that the relative cells viability was above 75% as compared to cell viability on a highly biocompatible glass slide (used as control). In this way, the qualitative analysis of the SEM micrographs, revealing the polygonal shape of the cells, indicating the viability of the cells, was confirmed quantitatively by MTS viability assay (Paun et al., 2019). Based on the fact that the microcage‐like structures developed and tested in this study were fabricated through the same method (LDW via TPP) and that the microcage‐like structures were made from the same material (IP‐L780/MNPs superparamagnetic composite) as the ones reported by us in the above mentioned studies, we can certainly state that the microcage‐like structures developed in the present work provide a highly biocompatible environment for osteoblast‐like cells and ensure a high cellular viability.

The interaction of living cells with magnetized biomaterial constructs has been previously studied and discussed (Wu et al., 2018). Thus, it has been reported that MNPs‐enriched polymeric matrices could be actuated through direct magnetic activation which has been shown to promote direct cell growth stimulation. This effect is done through the activation of mechanotransduction signaling pathways (Mannix et al., 2008; Eivazzadeh-Keihan et al., 2020), due to micromotions at the cell‐scaffold interface (Wu et al., 2018). Additionally, the incorporation of MNPs in the IP‐L780 photopolymer changes the topography through roughness enhancement (Ishmukhametov et al., 2022), which further increases the adhesion of cells and guides their growth, modulating the number of attachment points. Nevertheless, indirect stimulation comes from changes in the cells’ microenvironment due to external magnetic field stimulation (Wu et al., 2018).

The mechanism of bone cell stimulation in magnetically responsive structures is not yet well understood. It is believed that the MNPs withing the 3D microstructures, when they are activated‐exposed‐ to static magnetic fields, induce a micro‐deformation of the structures that further provides a strain stimulation to the cells (Aoki et al., 1990; Rosen, 1993; Kotani et al., 2000; Rosen, 2003; Bock et al., 2010; Bañobre-López et al., 2011; Meng et al., 2013). The strain stimulation would activate the cells to proliferate, differentiate and to form new bone tissue. The synergic effect of magnetically responsive biomimetic structures in response to the external applied magnetic field to fasten the osteogenesis can be amplified by physical cues that promote the cells adhesion. This can be achieved by fabricating 3D microstructures that are made by biocompatible materials with architectures that provide adhesive points for the cells (Paun et al, 2018b). Thus, the synergy between the architecture of the microcages’ 3D architecture and their actuation by static magnetic fields can act together to increase the number of adherent cells onto the structures, followed by differentiation and growth in 3D constructs similar with the natural tissues (Bock et al., 2010).

To conclude, in this study we demonstrated a proof of concept regarding osteoblast cells manipulation i.e., cells entrapment using 3D microcage‐like structures with superparamagnetic properties and actuated with static magnetic fields. The structures were fabricated using Laser Direct Writing via Two‐Photon Polymerization (LDW via TPP) of a composite material consisting of IP‐L780 biocompatible photopolymer mixed with iron oxide superparamagnetic nanoparticles (MNPs). The unique properties of LDW via TPP technique enabled the microcage‐like architecture of these 3D structures to be accurately reproduced, with a lateral resolution of about 90 nm. 3D hyperspectral microscopy provided evidence regarding the homogenous dispersion of the MNPs within the entire volume of the 3D structures. The superparamagnetic nature of the MNPs seeded with MG‐63 osteoblast‐like cells was proved through Scanning Electron Microscopy coupled with Energy Dispersive X‐Ray Spectroscopy. The comparative studies made on microcage‐like structures having superparamagnetic properties (i.e., made of IP‐L780/MNPs composite) and on similar structures made of IP‐L780 photopolymer alone (i.e., thus not having magnetic properties), indicated that the structures made by IP‐L780/MNPs composite that were actuated by static magnetic fields of 1.3 T were 13.66 ± 5.11 folds (*p* < 0.01) more efficient in terms of cells entrapment than the structures made by IP‐L780 photopolymer alone. The unique 3D architecture of the microcage‐like structures along with their superparamagnetic properties allowing their actuation by external static magnetic fields acted in synergy for efficient entrapping of osteoblast‐like cells, showing a significant potential for controlling cells adhesion and spreading and thus having a great potential for bone tissue engineering applications.

## Data Availability

The original contributions presented in the study are included in the article/Supplementary Material, further inquiries can be directed to the corresponding author.

## Supplementary material

SUPPLEMENTARY MOVIE S1 Tilted view obtained by enhanced dark field microscopy of a microcage‐like structure made of IP‐L780/MNPs composite. The microcage’s walls were colored in yellow and the centers of MNPs and MNPs aggregates were colored in red (false colors).
