# Supplementary figures and images for: Magnetically‐actuated microcages for cells entrapment, fabricated by laser direct writing via two photon polymerization
Source: Front Bioeng Biotechnol. 2023 Dec 19;11:1273277. doi: 10.3389/fbioe.2023.1273277 (PMC10758856; doi:10.3389/fbioe.2023.1273277)

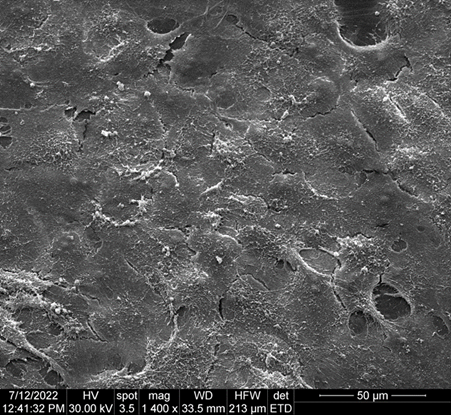

Supplement: Supplementary file 2 [file Image2.TIF]

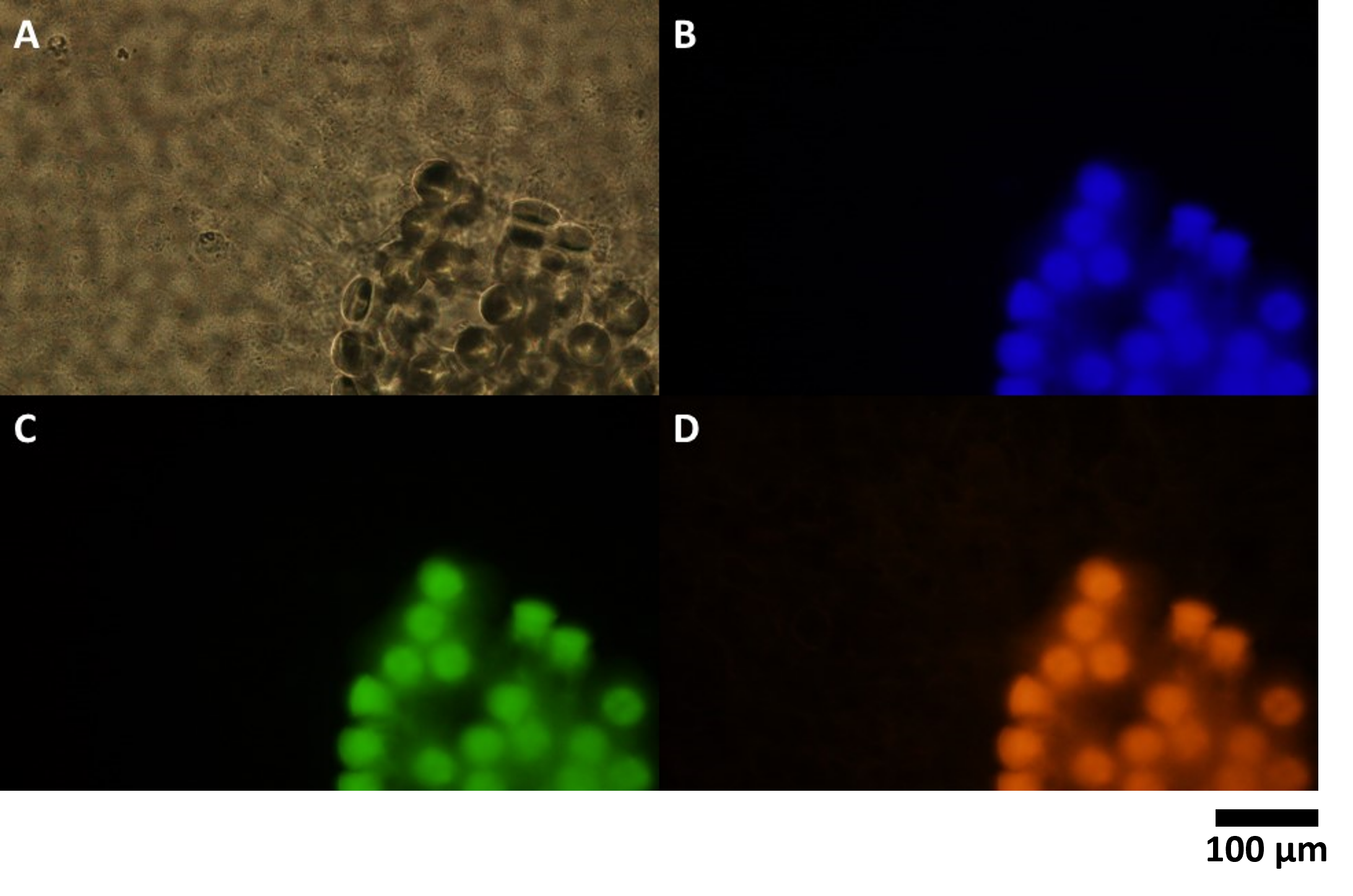

Supplement: Supplementary file 3 [file Image1.TIF]
